# Synthesis and Biological Evaluation of Novel Pyrimidine Amine Derivatives Bearing Bicyclic Monoterpene Moieties

**DOI:** 10.3390/molecules27228104

**Published:** 2022-11-21

**Authors:** Mingguang Zhang, Yunyun Wang, Shifa Wang, Hongyan Wu

**Affiliations:** 1 School of Pharmacology, Jiangsu Vocational College of Medicine, Yancheng 224005, China; 2 Jiangsu Province Key Laboratory for Inflammation and Molecular Drug Target, School of Pharmacy, Nantong University, Nantong 226001, China; 3 College of Chemical Engineering, Nanjing Forestry University, Nanjing 210037, China

**Keywords:** bicyclic monoterpene, pyrimidinamine derivatives, synthesis, antimicrobial activities, anti-inflammatory activity

## Abstract

A series of novel pinanyl pyrimidine amine derivatives (**1e**~**1n**) and camphoryl pyrimidine amine derivatives (**2b**~**2f**) bearing bicyclic monoterpene moieties were designed and synthesized from natural and renewable nopinone and camphor. All chemical structures of target compounds were characterized by ^1^H NMR, ^13^C NMR and HRMS spectra analyses, and the antimicrobial activities were evaluated. The results indicated that most compounds showed considerable antibacterial and antifungal activities against *Klebsiella pneumoniae*, *Streptococcus pneumoniae*, *Pseudomonas aeruginosa*, *Staphylococcus aureus*, *Escherichia coli*, *Methicillin-Resistant Staphylococcus aureus* (MRSA), *Bacillus cereus* and *Candida albicans.* Among them, **1f** showed potent antibacterial activity against all tested bacteria, **1i** exhibited excellent inhibition against *Streptococcus pneumoniae* (1 μg/mL) and *Escherichia coli* (1 μg/mL), which was better than the control drug amikacin (2 μg/mL). As to antifungal activity against *Candida albicans* (*C. albicans*), compound **1l** showed comparable activity (16 μg/mL) to the control drug ketoconazole. Furthermore, five active compounds with better antimicrobial activities also showed anti-inflammatory potencies against mouse mononuclear macrophages leukemia cells (RAW). Especially, **1f** (IC_50_ = 1.37 μM) and **2f** (IC_50_ = 1.87μM) are more potent than the control drug aspirin (IC_50_ = 1.91 μM).

## 1. Introduction

Heterocyclic compounds with nitrogen atoms were a vital class of organic compounds, most of them possessed various biological activities [1,2,3,4] and have been widely used in the medicine, pesticide, dye and food industries [5]. Pyrimidineamine derivatives characterized with two nitrogen atoms in the aromatic ring and a substituted amino outside showed valuable drug application, such as pyrimethamine (malaria prevention), imatinib (anti-tumor drug) and rosuvastatin (hyperlipidemic drug). Benefitting from the universal antibacterial and antiviral activities, tremendous novel pyridineamine compounds were developed in the pesticides, medicine and materials fields [6].

**Figure 1:**
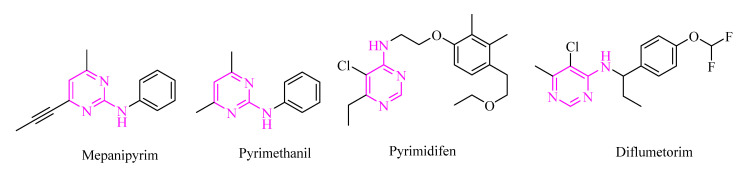
Structures of pyrimidine amine antibacterial agents.

As for the pesticides, pyrimidine amine fungicides showed antimicrobial potencies by inhibiting the biosynthesis of methionine and the secretion of cell wall-degrading enzymes in fungi, thereby inhibited the invasion of bacteria into host cells. Pyrimidine amine fungicides have excellent prevention and control effects on various diseases caused by botrytis cinerea with the characteristics of high activity, low toxicity and drug resistance. The marketed pyrimidine amine bacteriostatic agents, such as pyrimoxamine, pyrimydamine, pyrimoxetine and flufenoxamine [7], etc. (Figure 1), have been used to control botrytis, powdery mildew, scab, and rust infestation. In recent years, developing progress on new pyrimidine amine bacteriostatic agents have emerged one after another [8,9,10,11,12], which has been expected to become a new type of market bactericide after methoxyacrylate and succinate dehydrogenase. However, it is difficult to industrialize most pyrimidine amine compounds due to severe toxicity and heavy pollution [13], which has resulted to a low market competitiveness.

Methicillin-resistant Staphylococcus aureus (MRSA) is a ubiquitous bacterium pathogenic to humans and animals, a leading cause of infection worldwide, including a wide array of both hospital and community acquired infections [14]. Because of resistance to aminoglycosides, macrolides, tetracyclines, quinolones, sulfonamides and rifampicin at varying degrees, MRSA is currently called super bacteria, which could cause skin and soft tissue infections, bloodstream infections, and various organs infections of the body. This has led to critical global concerns. Candida species is a fungal pathogen extensively existed in the oral cavity, intestinal tract and upper respiratory tract that causes fungal vaginitis, thrush and other oral diseases, which threaten patients’ health and life [15]. *Candida albicans* can encounter commonly critically ill patients infected by an opportunistic fungal infection. The frequency of opportunistic *C. albicans* infections in critically ill patients ranked first among all fungal infection types. This led to patients in intensive care unit with prolonged stays due to coronavirus disease (COVID-19) is higher than in the period before COVID-19. Therefore, discovery of novel and effective antibacterial and antifungal agents with broad spectrum has attracted more and more attention [16].

Nopinone and camphor are natural and renewable resources, with good cell permeability, excellent biocompatibility and low toxicity, that display extensive antibacterial [17,18,19,20], antitumor [21,22], anti-inflammatory [23] and antiseptic [24] properties. Due to their unique bicyclic structures and exocyclic unsaturated carbonyl groups, nopinone and camphor are particularly reaction active that could be synthesized valuable compounds with rich structures through isomerization, reduction, condensation and esterification [25,26,27,28].

**Scheme 1:**
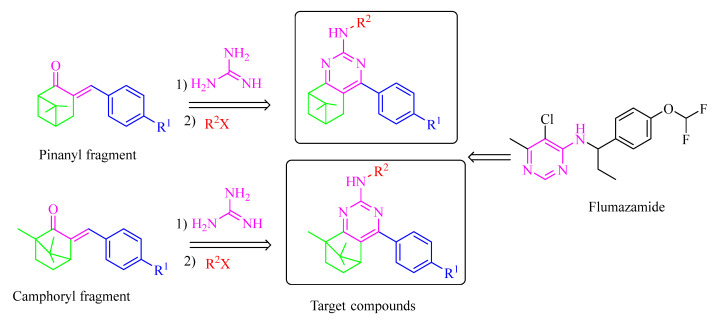
Design strategy of the target compounds.

**Scheme 2:**
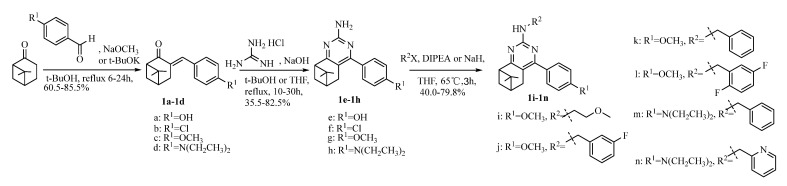
Synthesis of pinanyl pyrimidine amines derivatives with bicyclic monoterpene units.

In recent years, our research team has made some efforts to explore the application of pinane derivatives. Condensation of norpinone with thiosemicarbazide at exocyclic carbonyl group afforded norpinone thiosemicarbazone, then cyclized with α-halogenated ketone; new pinyl thiazole derivatives were obtained [29]. Biological evaluation results showed that these compounds showed certain insecticidal effect on *Lagerstroemia aphid* and displayed a concentration-dependent effect. In addition, insecticidal activities of nopinone-based pyrazole amides [30], antitumor activities of nopinone-based thiosemicarbazide derivative [31], amylase inhibition activities of nopinone-based thiazole hydrazones [32], and antitumor activities of camphor-based pyrimidine derivatives were also studied. These studies indicate that pinanyl heterocyclic derivatives have extensive biological activities, such as anticancer, anti-inflammatory, antioxidant, antibacterial, antidiabetic and antiviral, etc. Nevertheless, there are few studies reporting the synthesis of pyrimidine amine derivatives bearing bicyclic monoterpene moiety. The aforementioned findings give us an impetus to design and synthesize novel pyrimidine amines bearing bicyclic monoterpene moiety and explore their potential values as antimicrobial agents. Herein, we report the synthesis and biological evaluation of novel pinanylpyrimidine amines as antimicrobials. The design strategy of the target compounds is shown in Scheme 1:

Based on the above design strategy, a total of 15 novel pyrimidine amine derivatives with bicyclic monoterpene units were synthesized by the synthetic route shown in Scheme 2, and the structures of the compounds were characterized by ^1^H NMR, ^13^C NMR and HRMS. *K. pneumoniae*, *S. pneumoniae*, *P. aeruginosa*, *S. aureus*, *Escherichia coli* (*E. coli*), *methicillin-resistant Staphylococcus aureus* (MRSA), *Bacillus cereus* (*B. cereus*), and *Candida albicans* (*C. albicans*) were selected as test bacterial and fungi strains; the antibacterial activities and antifungal activity for the target compounds were evaluated. Antibacterial active compounds were selected for anti-inflammatory activity assay against mouse mononuclear macrophage leukemia cells (RAW), and the relationship between structures and activities were preliminarily discussed.

## 2. Results and Discussion

### 2.1. Chemistry

**Scheme 3:**
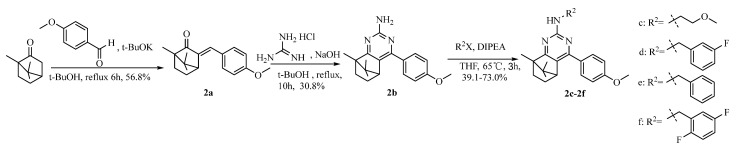
Synthesis of camphoryl pyrimidine amine derivatives with bicyclic monoterpene units.

The synthetic route for the target compounds is illustrated in Scheme 2 and Scheme 3. Briefly, compounds **1a**~**1d** were synthesized with a typical aldehyde-ketone condensation reaction [33]. The *α*-C of nopinone was converted to carbanion with the catalyst of alkali, then an addition reaction was performed between the carbanion and aldehyde carbonyl to generate *α*, *β* -unsaturated ketones. Based on the principle, nopinone as the starting material, condensed with *p*-hydroxybenzaldehyde, *p*-chlorobenzaldehyde, *p*-methoxybenzaldehyde and *p*- diethylaminobenzaldehyde, pinanyl ketene intermediates (**1a**~**1d**) were generated in the presence of sodium methoxide or potassium tert-butoxide.

Since the strong electrondonating effects of methoxy and diethylamine groups, the reactions of **1c** and **1d** were difficult and sodium methoxide were used instead of potassium tert-butoxide, and the reaction time were extended to 16~24 h with the yields of 84~86%. **1a**~**1c** were purified by recrystallization from ethanol as solid products, **1d** was oily product with diethylamino group, which was purified by column chromatography. Pinanyl pyrimidines (**1e**~**1h**) were obtained by cyclization **1a**~**1d** with guanidine hydrochloride in the presence of sodium hydroxide. For the synthesis of **1g**, the reaction time was long (30 h) and the yield was relatively low (35.5%) because of the methoxy deactivation effects. Pinanyl pyrimidine amines (**1i**~**1n**) were generated by further substitution of **1g** or **1h** with haloalkanes. The products were poorly dissolved in dichloromethane, ethanol and acetonitrile, and the reaction hardly occurred, thus the suitable solvent tetrahydrofuran was explored. For most instances, the substituted amination of haloalkanes and aminopyrimidines could react smoothly catalyzed by DIPEA. However, for preparing **1i** by amination of 2-bromoethyl methyl ether and **1g**, the reaction was difficult. A stronger base sodium hydride was used at 65 °C for 3 h, and the yield of the product was 79.8%. As for camphor, condensation of camphor and *p*-methoxybenzaldehyde occurred in tert-butanol solvent under the catalysis of potassium tert-butoxide, and the intermediate **2a** was generated, followed by cyclization with guanidine hydrochloride, the camphorylpyrimidine intermediate **2b** was afforded. According to the preparation method of pinanyl pyrimidineamines, camphoryl pyrimidineamine compounds (**2c**~**2f**) were obtained by substitution of **2b** with alkyl halides in tetrahydrofuran solvent.

### 2.2. Structural Characterization of Compounds

The characterization of target compounds mainly focused on the presence of pinanyl, camphoryl and pyrimidineamine functional groups in the structures.

For the pinanyl pyrimidine amines, in the ^1^H NMR spectrums, chemical shifts on the saturated carbons atoms of benzylidene nopinone intermediates were in the high fields (*δ* 0.81–2.99), including 6 H of methyl group and 6 H in the pinene ring. Hydrogen atom chemical shifts on the unsaturated carbons atoms appeared in the low fields with signals at *δ* 6.68–7.69 that corresponded to 4 H on the benzene ring and 1 H in the double bond of the pinene. For pinanyl pyrimidine amine products, the chemical shifts on the saturated carbons appeared in the range of *δ* 0.62–2.96, while those on benzene rings and pyridine rings appeared in the low fields with signals at *δ* 6.13–8.5. In the ^13^C NMR spectrums, the chemical shifts (*δ*) of the saturated carbons on the pinene ring appeared in high fields at *δ* 21.00–55.89, while those of unsaturated carbons on benzene, pyridine or pyrimidine rings appeared in low fields at *δ* 103.86–176.13. Because of the deshielding effects of C=O, chemical shifts of carbonyl carbon atoms were shifted to the lower fields at *δ* 201.54–203.85. Owing to the influences of fluorine atoms, F-C coupling phenomenon was observed in the ^13^C NMR spectra of compounds **1j** and **1l**. For compound **1j**, fluorine atom coupled with three carbon atoms (δ 161.60, δ 137.58 and 115.22) on the fluorine-containing benzene ring, resulted two signals for every carbon atom.

For the camphorylpyrimidine amines, in the ^1^H NMR spectrums, chemical shifts on the saturated carbons atoms of benzylidene camphorone intermediates were in the high fields (*δ* 0.83–3.11) including 9 H of methyl group and 5 H in the camphor ring. Hydrogen atom chemical shifts on the unsaturated carbons atoms appeared in the low fields with signals at *δ* 6.93–7.48, that corresponded to 4 H on the benzene ring and 1 H in the double bond of the olefin. For camphoryl pyrimidine amine products, the chemical shifts on the saturated carbons appeared in the range of *δ* 0.54–3.08, while those on benzene rings and pyridine rings appeared in the low fields with signals at *δ* 6.26–7.80. In the ^13^C NMR spectrums, the chemical shifts (*δ*) of the saturated carbons on the camphor ring appeared in high fields at *δ* 10.04–71.65, while those of unsaturated carbons on the benzene, pyridine or pyrimidine rings appeared in low fields at *δ* 103.70–181.81. Affected by C=O, chemical shifts of carbonyl carbon atoms were shifted to the lower fields, and the F-C coupling phenomenons were also observed in the ^13^C NMR spectra of compounds **2d** and **2f**. The coupling splitting in the ^13^C NMR spectrum indicated the presence of fluorine atoms, which were consistent with the structures.

In the HRMS spectrums, the molecular ion peaks of the target compounds were consistent with the theoretical value with the deviation below 0.5%, which furtherly confirmed the structures of the target compounds.

### 2.3. Biological Evaluation

#### 2.3.1. Antibacterial Activity Assay

Clinical isolates of MRSA are susceptible to amikacin at concentrations achieved by regional perfusion, amikacin was chosen to be the reference substance. The antibacterial activities of compounds **1e**~**1n** and **2b**~**2f** in vitro were evaluated by MIC assay with double dilution method [34]. The selected strains included *K. pneu-moniae*, *S. pneumoniae*, *Pseudomonas aeruginosa P. aeruginosa*, *S. aureus*, *E. coli*, *MRSA, B. cereus* and *white Candida (C. albicans)*; the biological activity assay results are shown in Table 1:

As shown in Table 1, most compounds showed moderate to excellent antibacterial activities against certain bacteria, while **1e**, **1f**, **1g**, **1i** and **2f** exhibited favorable inhibition to most bacteria. Among them, **1i** displayed excellent antibacterial activities against *Streptococcus pneumoniae (S. pneumoniae)* and *Escherichia coli (E. coli)* with MIC value of 1 μg/mL, which was superior to amikacin. **1e**, **1f** and **2f** inhibited *K*. *pneumoniae* with MIC value of 32 μg/mL that was comparable to amikacin. **1f** displayed better antibacterial effect against *P. aeruginosa* (16 μg/mL) than amikacin (32 μg/mL). **1f**, **1g**, **1h**, **1l** and **2f** displayed weaker antibacterial effect against *MRSA* (8 μg/mL) than amikacin (1 μg/mL). As for antifungal activities, compound **1l** exhibited the best activity against *C. albicans* with MIC value of 16 μg/mL, which was comparable to ketoconazole.

Preliminary structure–activity relationship analysis for compounds **1e**~**1n** and **2b**~**2f** showed that when R^2^ was H, the target compounds had a certain inhibitory activity against most bacteria, but no inhibitory activity against fungi when R^2^ was alkyl or aryl groups, compounds showed different antibacterial activities against bacteria and fungi. These results indicated the introduction of an electron-withdrawing group with F (such as **2f**) would likely keep the antibacterial potencies while the introduction of an electron-donating group with diethylamine would decrease the antibacterial activities. From the data in Table 1, it can be seen that the antibacterial MIC values of **1i** and **1f** were lower than that of amikacin; the antifungal MIC of **1l** was comparable to ketoconazole, which have potential applications in antimicrobial drug research.

So far, five compounds were determined with effective antibacterial activities. Especially, the promising compound **1i** displayed better antibacterial activities against *Streptococcus pneumoniae (S. pneumoniae)* and *Escherichia coli (E. coli)* than amikacin. In addition, compound **1l** exhibited comparable antifungal activity to ketoconazole. The properties of chemical structures, such as molecule weight, CLogP values, pKa values and tPSA values, affect lipophilicity, solubility, protein binding and the ability of a chemical to cross the cell membrane, thus governing the absorption, distribution, metabolism, excretion and toxicity. In order to illustrate the structure characters of active compounds, the properties comparison of compounds **1e**, **1f**, **1g**, **1i**, **2f** and **1l** were summarized in Table 2.

The data indicated that all the active compounds have smaller molecule weights than the control drugs, the predicted CLogP values were less than 7.26 and the predicated tPSA values were above 45.98 for all target compounds. As to antibacterial activities, compounds **1i** (CLogP = 5.01, tPSA = 55.21) displayed excellent potencies with higher CLogP and lower tPSA than amikacin (CLogP = −4.12, tPSA = 331.94). For the available Pka values, amikacin is water-soluble with higher Pka and tPSA values but a lower CLogP value. As to the promising compound **1l**, compared with ketoconazole (CLogP = 3.64, tPSA = 66.84), **1l** (CLogP = 6.74, tPSA = 45.98) displayed similar antifungal potent to *C. albicans* with higher CLogP and lower tPSA value.

#### 2.3.2. Anti-Inflammatory Activity Assay

To characterize whether compounds we synthesized can induce inflammatory cell apoptosis, the MTT assay was performed on the RAW cells. Based on the preliminary screening of antibacterial activity, five compounds (**1e**, **1f**, **1g**, **1i** and **2f**) with outstanding antibacterial effects were selected for anti-inflammatory activity assay, and aspirin was used as the positive control drug. The anti-inflammatory activities of the five target compounds on mouse mononuclear macrophage leukemia cells (RAW) are shown in Table 3.

From the results in Table 3, it can be seen that the five compounds **1e**, **1f**, **1g**, **1i** and **2f** have obvious anti-inflammatory activities on RAW cells, especially, **1f** (IC_50_ = 1.37μM) and **2f** (IC_50_ = 1.87 μM) displayed better inhibition than the control aspirin (IC_50_ = 1.91 μM). Structure–activity relationship analysis: substituted amino R^2^ has a great influence on the anti-inflammatory activity. While the electron withdrawing group (F) was introduced into the benzene para position, such as **2f**, it is beneficial to maintain the anti-inflammatory activity. An electron-donating group such as ethoxymethyl was introduced to the substituted amino group; the anti-inflammatory activity was decreased.

#### 2.3.3. Molecular Docking Study

**Figure 2:**
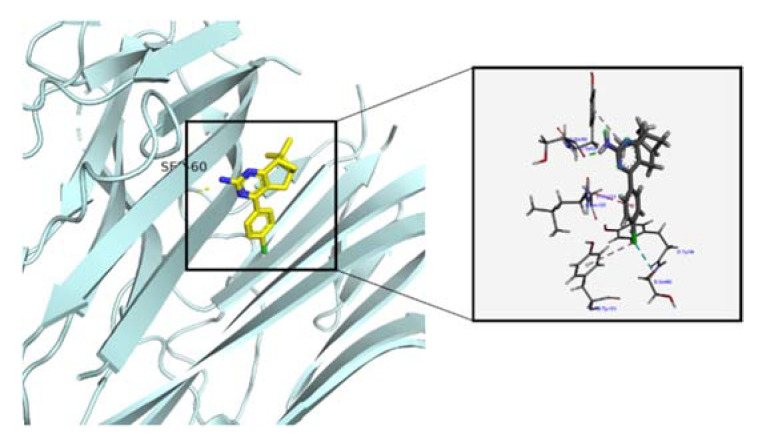
The binding mode of compound **1f** with inflammation protein (PDB ID: 2AZ5).

To explore the binding mode of compounds **1f** with inflammation protein (PDB ID: 2AZ5), Auto Dock software was used to predict the putative binding modes. As shown in Figure 2, it can be seen that compound **1f** bound to the active pocket of tumor necrosis factor protein, fitted well to the active pocket of the protein. The chlorophenyl ring fragment penetrated deep into the active cavity of the protein while the pyrimidine ring embedded in the upper active cavity of the TNF protein. Further analysis of the binding mode with amino acid residue in the protein pocket showed that nitrogen atoms in pyrimidine ring and chlorine atom in chlorobenzene unit formed hydrogen bonds with Ser60. In addition, the pyrimidine ring and the phenyl ring formed H–π interactions with Tyr59 and Gyl121, respectively. Compared with aspirin scored of −8.1, further flexible docking results indicated there is a strong biding ability between compound **1f** and the TNF protein with the score of −7.9.

## 3. Conclusions

In summary, from natural and renewable products nopinone and camphor, a series of novel pinanyl pyrimidine amine and camphoryl pyrimidine amine derivatives were designed and synthesized; their antibacterial activity and anti-inflammatory activity in vitro were evaluated. Experimental results showed that most of the compounds showed broad-spectrum antibacterial activity against several bacterial species. Among them, compound **1i** exhibited better inhibitory activity against *Streptococcus pneumoniae* (1 μg/mL) and *Escherichia coli* (1 μg/mL) than control amikacin (2 μg/mL), compound **1l** showed excellent antifungal activity with the lowest inhibitory concentration of 16 μg/mL, which is comparable to ketoconazole. Five compounds with better antibacterial activity also showed obvious anti-inflammatory activities against RAW cells. Especially, compound **1f** (IC_50_ = 1.37 μM) and **2f** (IC_50_ = 1.87 μM) exhibited better anti-inflammatory activities than that of control aspirin (IC_50_ = 1.91 μM). Molecular docking study exhibited that **1f** could bind tightly to the inflammation protein that was consistent with the anti-inflammatory activity. All in all, it suggested that the target pyrimidine amines were expected to be a potential bacteriostat or anti-inflammatory agent, which is worthy of further study.

## 4. Experimental Section

### 4.1. Chemistry

All reagents and solvents were purchased from commercial sources and used without further purification. Flash chromatography was performed using silica gel with 200–300 mesh produced by Qingdao Ocean Chemical Factory. All reactions and processes of flash chromatography were monitored by the TLC method using silica gel plates with fluorescence F254 and iodine visualization. The melting points were determined with a YRT-3 drug melting point instrument produced by Tianjin Tianda Tianfa Technology Co., Ltd., and the thermometer was not calibrated. High-resolution mass spectrometry was analyzed by an Applied Biosystems Q-STAR Elite ESI-LC-MS/MS mass spectrometer. ^1^H NMR and ^13^C NMR spectra were recorded on a Brucker AV- 400 spectrometer at 400 MHz and Brucker AV-500 spectrometer at 125 MHz using CDCl_3_ and DMSO-d_6_ as solvents, TMS as internal standard (Supplementary Materials). Coupling constants (*J*) are expressed in hertz (Hz). Chemical shifts (δ) are given in parts per million (ppm). The purity of the compounds was determined by Agilent 1260 liquid chromatograph fitted with an Inertex-C18 column. All target compounds have purity over 95%.

#### 4.1.1. Synthesis of Intermediate 3-Arylbenzylidene Nopinones **1a**~**1d**

In a solution of tert-butanol (30 mL) and norpinone (14.48 mmol), *p*-hydroxybenzaldehyde (14.48 mmol) and sodium methoxide (57.93 mmol) were slowly added under stirring. After feeding completed, the reaction mixture was refluxed for 6–7 h, the solvents were concentrated under reduced pressure, water (25 mL) was added to the residue, the reaction mixture was extracted with ethyl acetate (15 mL × 3), and the organic layers were washed with saturated brine to neutrality. After solvent evaporation, the resulting crude product was recrystallized from methanol to give **1a**. Intermediates **1b** to **1d** were prepared by the same method.

*3-(4’-Hydroxybenzylidene)nopinone* (**1a**): pale yellow crystal solid, yield: 76.7%, melting point: 206–207 °C. ^1^H NMR (400 MHz, DMSO-*d*_6_, ppm) *δ*: 10.00 (s, 1 H, Ar-OH), 7.52 (d, *J* = 8.6 Hz, 2 H, ArH), 7.49 (s, 1 H, ArCH = C), 6.86 (d, *J* = 8.6 Hz, 2 H, ArH), 2.94–2.81 (m, 2 H), 2.59 (dt, *J* = 10.7, 5.8 Hz, 1 H), 2.51 (t, *J* = 5.5 Hz, 1 H), 2.36–2.25 (m, 1 H), 1.37 (d, *J* = 10.2 Hz, 1 H), 1.32 (s, 3 H, CH_3_), 0.81 (s, 3 H, CH_3_). ^13^C NMR (100 MHz, DMSO-*d*_6_, ppm) *δ*: 201.71, 158.60, 134.67, 132.78, 129.18, 126.25, 115.69, 55.14, 40.15, 30.53, 27.02, 25.83, 21.29. HRMS-ESI: 243.1385 calcd for C_16_H_19_O_2_ [M+H]^+^, found: 243.1386.

*3-(4′-Chlorobenzylidene)nopinone* (**1b**): The synthetic method of **1a** was taken as reference, nopinone and *p*-chlorobenzaldehyde were selected as raw material, a light yellow crystal solid was obtained with the yield of 60.5%, melting point: 107–109 °C. ^1^H NMR (400 MHz, DMSO-*d*_6_, ppm) *δ*: 7.68 (d, *J* = 8.6 Hz, 2 H, ArH), 7.53 (s, 1 H, ArCH=C), 7.50 (d, *J* = 8.6 Hz, 2 H, ArH), 2.92 (t, *J* = 2.8 Hz, 2 H), 2.55–2.59 (m, 1 H), 2.28–2.33 (m, 1 H), 1.39 (d, *J* = 10.3 Hz, 1 H), 1.33 (s, 3 H, CH_3_), 1.26 (d, *J* = 19.9 Hz, 1 H), 0.82 (s, 3 H, CH_3_). ^13^C NMR (100 MHz, DMSO-*d_6_*, ppm) *δ*: 201.54, 133.94, 133.67, 133.51, 133.07, 132.27, 128.68, 55.20, 40.34, 38.76, 30.29, 26.83, 25.80, 21.33. HRMS-ESI: 261.1046 calcd for C_16_H_18_ClO [M+H]^+^, found: 261.1043.

*3-(4-Methoxybenzylidene)-nopinone* (**1c**): The synthetic method of **1a** was taken as reference, nopinone and *p*-methoxybenzaldehyde were selected as raw material, a light yellow crystal solid was obtained with the yield of 85.5%, melting point: 71–72 °C. ^1^H NMR (400 MHz, CDCl_3_, ppm) *δ*: 7.69 (t, *J* = 2.0, 2.4 Hz, 1 H, ArH), 7.59 (t, *J* = 2.0, 6.8 Hz, 2 H, ArH, ArCH=C), 6.95–6.98 (m, 2 H, ArH), 3.87 (s, 3 H, OCH_3_), 2.98 (t, *J* = 2.4, 3.2 Hz, 2 H), 2.71–2.72 (m, 1 H), 2.61–2.69 (m, 1 H), 2.37–2.41 (m, 1 H), 1.53 (d, *J* = 10.4 Hz, 1 H), 1.40 (s, 3 H, CH_3_), 0.95 (s, 3 H, CH_3_); ^13^C NMR (100 MHz, CDCl_3_, ppm) *δ*: 203.59, 160.21, 135.43, 132.61, 130.33, 128.56, 114.14, 55.89, 55.34, 40.83, 39.53, 31.04, 27.60, 26.25, 21.64. HRMS-ESI: 257.1542 calcd for C_17_H_21_O_2_ [M+H]^+^, found: 257.1543.

*3-(4’-N,N-diethylaminobenzylidene) nopinone* (**1d**): The synthetic method of **1a** was taken as reference, nopinone and *p*-diethylaminobenzaldehyde were selected as raw material, light yellow oil was obtained after column chromatography purification with the yield of 84.6%. ^1^H NMR (400 MHz, CDCl_3_, ppm) *δ*: 7.65 (s, 1 H, ArCH=C), 7.52 (d, *J* = 8.9 Hz, 2 H, ArH), 6.68 (d, *J* = 8.9 Hz, 2 H, ArH), 3.40 (q, *J* = 14.1, 7.0 Hz, 4 H, NCH_2_), 2.90–2.99 (m, 2 H), 2.65 (t, *J* = 5.6 Hz, 1 H), 2.35–2.37 (m, 1 H), 1.50 (d, *J* = 10.2 Hz, 1 H), 1.37 (d, *J* = 3.36 Hz, 4 H, CH_3_, CH), 1.19 (t, *J* = 7.1 Hz, 6 H, CH_3_), 0.92 (s, 3 H, CH_3_). ^13^C NMR (100 MHz, CDCl_3_, ppm) *δ*: 203.85, 148.18, 136.40, 133.11, 127.12, 122.89, 111.17, 55.87, 44.41, 40.67, 39.70, 27.84, 26.28, 21.61, 12.62. HRMS (*m*/*z*): 298.2171 calcd for C_20_H_28_NO [M +H]^+^, found: 298.2167.

#### 4.1.2. Synthesis of Pinanealkylpyrimidineamines **1e**~**1n**

To a 50 mL dry three-necked flask equipped with a thermometer and a condenser, tert-butanol (25 mL), **1a** (4.1 mmol), guanidine hydrochloride (6.2 mmol) were added in sequence; the mixture of sodium hydroxide (7.8 mmol) and water (10 mL) was added below 50 °C. The reaction mixture was refluxed for 10 h then extracted three times with ethyl acetate (10 mL × 3), the combined organic layers were washed with saturated brine until neutral, the solvent was removed under reduced pressure. The residue was purified by column chromatography (petroleum ether/ethyl acetate = 3/1) to give a yellow solid of pure **1e**. Compounds **1f**~**1h** were prepared in the same way as **1e**.

*7,7-Dimethyl-5,6,7,8-tetrahydro-4-(4’-hydroxyphenyl)-6,8-methylene-2-quinazolinamine* (**1e**): light yellow powder, yield: 82.5%, melting point: 271–273 °C. ^1^H NMR (400 MHz, DMSO-*d*_6_, ppm) *δ*: 7.50 (d, *J* = 8.7 Hz, 2 H, ArH), 6.83 (d, *J* = 8.6 Hz, 2 H, ArH), 4.00 (s, 2 H, NH_2_), 2.61–2.81 (m, 2 H), 2.50–2.59 (m, 2 H), 2.24–2.26 (m, 1 H), 1.30 (s, 3 H, CH_3_), 1.15 (d, *J* = 9.4 Hz, 1 H), 0.62 (s, 3 H, CH_3_). ^13^C NMR (100 MHz, DMSO-*d*_6_, ppm) *δ*: 176.09, 162.95, 161.03, 158.06, 130.49, 129.58, 115.29, 112.63, 49.76, 40.27, 29.74, 29.63, 25.83, 21.33. HRMS (*m*/*z*): 282.1606 calcd for C_17_H_20_N_3_O [M+H]^+^, found: 282.1605.

*7,7-Dimethyl-5,6,7,8-tetrahydro-4-(4’-chlorophenyl)-6,8-methylene-2-quinazolinamine* (**1f**): the synthetic method of **1e** was taken as reference, **1b** was selected as raw material, a off-white solid was obtained with the yield of 80.1%, melting point: 161–164 °C. ^1^H NMR (400 MHz, DMSO-*d*_6_, ppm) *δ*: 7.95 (d, *J* = 8.5 Hz, 1 H, ArH), 7.69 (d, *J* = 8.5 Hz, 1 H, ArH), 7.57 (d, *J* = 8.6 Hz, 1 H, ArH), 7.51 (d, *J* = 8.4 Hz, 1 H, ArH), 6.36 (s, 2 H, NH_2_), 2.81 (dd, *J* = 16.4, 3.1 Hz, 1 H), 2.70 (dd, *J* = 16.4, 2.6 Hz, 1 H), 2.65 (t, *J* = 5.5 Hz, 1 H), 2.62–2.59 (m,1 H), 2.31–2.28 (m, 1 H), 1.36 (s, 3 H, CH_3_), 1.24 (d, *J* = 8.4 Hz, 1 H), 0.69 (s, 3 H, CH_3_). ^13^C NMR (100 MHz, DMSO-*d*_6_, ppm) *δ*: 176.13, 166.92, 161.87, 161.69, 138.24, 137.94, 133.86, 131.60, 130.78, 130.16, 129.20, 128.58, 112.34, 50.06, 38.69, 29.81, 29.27, 26.09, 21.56. HRMS-ESI: 300.1268 calcd for C_17_H_19_ClN_3_ [M+H]^+^, found: 300.1271.

*7,7-Dimethyl-4-(4-methoxyphenyl)-5,6,7,8-tetrahydro-6,8-methylenequinazolin-2-amine* (**1g**): the synthetic method of **1e** was taken as reference, **1c** was selected as raw material, a white solid was obtained with the yield of 35.5%, melting point: 173–175 °C. ^1^H NMR (400 MHz, DMSO-*d_6_*, ppm) *δ*: 0.65 (s, 3 H, CH_3_), 1.22–1.24 (m, 1 H), 1.34 (s, 3 H, CH_3_), 2.27–2.30 (m, 1 H), 2.58–2.60 (m, 1 H), 2.61–2.66 (m, 1 H), 2.71–2.76 (m, 1 H), 2.83–2.87 (m, 1 H), 3.80 (s, 3 H, OCH_3_), 6.21 (s, 2 H, NH_2_), 7.00 (dd, *J* = 2.8, 8.8 Hz, 2 H, ArH), 7.64–7.68 (m, 2 H, ArH), ^13^C NMR (100 MHz, DMSO-*d_6_*, ppm) *δ*: 175.72, 162.40, 161.82, 160.05, 131.50, 130.44, 113.84, 111.99, 55.62, 50.07, 38.61, 29.88, 29.73, 26.08, 21.51. HRMS-ESI: 296.1763 calcd for C_18_H_22_N_3_O [M+H]^+^, found: 296.1765.

*7,7-Dimethyl-5,6,7,8-tetrahydro-4-(4’-N,N-diethylaminophenyl)-6,8-methylene-2-quinazole Linoamine* (**1h**): the synthetic method of **1e** was taken as reference, **1d** was selected as raw material, a pale yellow solid was obtained with the yield of 78.5%, melting point: 146–148 °C. ^1^H NMR (400 MHz, DMSO-*d*_6_, ppm) *δ*: 7.63 (d, *J* = 8.0 Hz, 2 H, ArH), 6.68 (d, *J* = 8.0 Hz, 2 H, ArH), 6.13 (s, 2 H, NH_2_), 2.96–2.74 (m, 2 H), 2.70–2.54 (m, 2 H), 2.40 (d, *J* = 81.4 Hz, 2 H), 1.34 (s, 4 H, CH_3,_ CH), 1.22 (d, *J* = 7.5 Hz, 2 H), 1.11 (t, *J* = 6.0 Hz, 7 H), 0.67 (s, 3 H, CH_3_). ^13^C NMR (100 MHz, DMSO-*d*_6_, ppm) *δ*: 175.26, 162.36, 161.64, 148.16, 130.50, 125.35, 111.39, 110.81, 50.07, 44.09, 38.54, 30.29, 29.92, 26.09, 21.49, 12.92. HRMS-ESI: 337.2392 calcd for C_21_H_29_N_4_ [M+H]^+^, found: 337.2390.

#### 4.1.3. Synthesis of Pinanylpyrimidinamines **1i**~**1n**

To a solution of THF (10 mL) and compound **1g** (1.02 mmol), NaH (4.06 mmol) was added in portions below 0 °C with the protection of N_2_, the solution was stirred for another 30 min, then 2-bromoethylmethyl ether (1.12 mmol) was added dropwise within 10 min and the reaction mixture was stirred at 65 °C for 3 h. After cooling to room temperature, the reaction mixture was poured into ice water and extracted with EtOAc (2 × 20 mL). The combined organic layers were washed with brine and dried over Na_2_SO_4_; after filtered, the solvent was removed under reduced pressure. The residue was purified by column chromatography (petroleum ether/ethyl acetate = 4/1) to give a white solid of pure **1i**. Compounds **1j**~**1n** were prepared by the same method as **1i**.

*7,7-Dimethyl-N-(2-methoxyethyl)-4-(4-methoxyphenyl)-5,6,7,8-tetrahydro-6,8-methylene quinazolin-2-amine* (**1i**): the target compound was obtained as off-white solid with the yield of 79.8%, melting point: 125–127 °C. ^1^H NMR (400 MHz, DMSO-*d_6_*, ppm) *δ*: 0.69 (s, 3 H, CH_3_), 1.23 (d, *J* = 9.2, 1 H), 1.36 (s, 3 H, CH_3_), 2.29–2.32 (m, 1 H), 2.51–2.67 (m, 2 H), 2.75–2.91 (m, 2 H), 3.26 (s, 3 H, OCH_3_), 3.46–3.47 (m, 4 H), 3.82 (s, 3 H, OCH_3_), 6.67–6.69 (m, 1 H, NH), 7.01–7.04 (m, 2 H, ArH), 7.01–7.04 (d, *J* = 8.4, 2 H, ArH), ^13^C NMR (100 MHz, DMSO-*d_6_*, ppm) *δ*: 21.55, 26.07, 29.78, 29.87, 38.61, 50.25, 55.66, 58.35, 71.20, 111.88, 113.93, 130.48, 131.65, 160.13, 160.60, 175.68. HRMS-ESI: 354.2182 calcd for C_21_H_28_N_3_O [M+H]^+^, found: 354.2183.

*N-(3-Fluorobenzyl)-4-(4-methoxyphenyl)-7,7-dimethyl-5,6,7,8-tetrahydro-6,8-methylenequinoline Oxazolin-2-amine* (**1j**): the synthetic method of **1i** was taken as reference, **1g** and 1-bromomethyl-3-fluorobenzene were selected as raw materials, a white solid was obtained with the yield of 66.4%. Melting point: 112–115 °C. ^1^H NMR (400 MHz, DMSO-*d_6_*, ppm) *δ*: 7.68 (d, *J* = 2.0, 6.8 Hz, 2 H, ArH), 7.34–7.39 (m, 3 H, ArH), 7.09–7.14 (m, 2 H, ArH), 7.00 (dd, *J* = 2.0, 7.2 Hz, 1 H, ArH), 4.48–4.50 (m, 2 H), 3.81 (s, 3 H, OCH_3_), 2.79–2.86 (m, 2 H), 2.50–2.67 (m, 2 H), 2.29–2.30 (m, 1 H), 1.35 (s, 3 H, CH_3_), 1.23 (d, *J* = 9.3, 1 H), 0.68 (s, 3 H, CH_3_). ^13^C NMR (100 MHz, DMSO-*d_6_*, ppm) *δ*: 175.79, 161.60 (d, *J* = 216.3 Hz), 160.27, 160.15, 137.58 (d, *J* = 3.0 Hz). 131.59, 130.51, 129.67, 129.59, 115.22 (d, *J* = 21 Hz), 113.91, 112.14, 55.64, 50.26, 43.93, 40.66, 29.84, 29.81, 26.05, 21.53. HRMS-ESI: 404.2138 calcd for C_25_H_27_FN_3_O [M+H]^+^, found: 404.2132.

*N-benzyl-4-(4-methoxyphenyl)-7,7-dimethyl-5,6,7,8-tetrahydro-6,8-methylenequinazoline-2- Amine* (**1k**): the synthetic method of **1i** was taken as reference, **1g** and benzyl chloride were selected as raw materials, a yellow solid was obtained with the yield of 65.1%, melting point: 128–131 °C. ^1^H NMR (400 MHz, DMSO-*d_6_*, ppm) *δ*: 7.69 (d, *J* = 8.8 Hz, 2 H, ArH), 7.34–7.36 (m, 3 H, ArH), 7.30 (t, *J* = 7.5 Hz, 2 H, ArH), 7.21 (m, 1H, ArH), 7.00 (d, *J* = 8.8 Hz, 2 H, ArH, NH), 4.51–4.53 (m, 2 H), 3.80 (s, 3 H, OCH_3_), 2.79–2.90 (m, 2 H), 2.67 (t, *J* = 5.5 Hz, 1 H), 2.57–2.62 (m, 1 H), 2.28–2.32 (m,1 H), 1.35 (s, 3 H, CH_3_), 1.23 (d, *J* = 9.4, 1 H), 0.69 (s, 3 H, CH_3_). ^13^C NMR (100 MHz, DMSO-*d_6_*, ppm) *δ*: 175.74, 160.61, 160.13, 141.43, 131.61, 130.51, 128.53, 127.73, 126.84, 113.90, 112.03, 55.64, 50.26, 44.61, 29.86, 29.82, 26.06, 21.54. HRMS-ESI: 386.2232 calcd for C_25_H_28_N_3_O [M+H]^+^, found: 386.2227.

*N-(2,4-Difluorobenzyl)-4-(4-methoxyphenyl)-7,7-dimethyl-5,6,7,8-tetrahydro-6,8-diode Methylquinazolin-2-amine* (**1l**): the synthetic method of **1i** was taken as reference, **1g** and 2,4-difluorobenzyl bromide were selected as raw materials, a gray solid was obtained with the yield of 40.0%, melting point: 125–126 °C. ^1^H NMR (400 MHz, DMSO-*d_6_*, ppm) *δ*: 7.68 (dd, *J* = 2.0, 6.8 Hz, 2 H, ArH), 7.44 (q, *J* = 6.8 Hz, 1 H, ArH), 7.34 (t, *J* = 6.6 Hz, 1 H, ArH), 7.15–7.20 (m, 1 H, ArH), 6.99–7.03 (m, 3 H, ArH, NH), 4.52–4.53 (m, 2 H), 3.81 (s, 3 H, OCH_3_), 2.80–2.87 (m, 2 H), 2.67 (t, *J* = 5.6 Hz, 1 H), 2.51–2.59 (m, 1 H), 2.28–2.32 (m, 1 H), 1.35 (s, 3 H, CH_3_), 1.23 (d, *J* = 9.2, 1 H), 0.68 (s, 3 H, CH_3_). ^13^C NMR (100 MHz, DMSO-*d_6_*, ppm) *δ*: 175.86, 161.59 (d, *J* = 256.5 Hz), 162.73, 160.54 (d, *J* = 258.5 Hz), 161.70, 160.39, 160.19, 131.50, 131.16, 131.07 (d, *J* = 3.0 Hz), 131.00, 130.52, 124.26 (dd, *J* = 15.0, 3.0 Hz), 113.92, 112.40, 111.50 (dd, *J* = 20.9, 3.6 Hz), 103.86 (t, *J* = 25.6 Hz), 55.65, 50.25, 38.61, 37.97 (d, *J* = 3.9 Hz), 29.83, 26.04, 21.52. HRMS-ESI: 422.2044 calcd for C_25_H_26_F_2_N_3_O [M+H]^+^, found: 422.2046.

*N-benzyl-4-(4-(diethylamino)phenyl)-7,7-dimethyl-5,6,7,8-tetrahydro-6,8-methylquinazoline-2- Amine* (**1m**): the synthetic method of **1i** was taken as reference, **1f** and benzyl chloride were selected as raw materials, a yellow white solid was obtained with the yield of 46.5%, melting point: 57–60 °C. ^1^H NMR (400 MHz, DMSO-*d*_6_, ppm) *δ*: 7.66 (d, *J* = 8.6 Hz, 2 H, ArH), 7.28–7.36 (m, 4 H, ArH), 7.18–7.22 (m, 2 H, ArH), 6.68 (d, *J* = 8.6 Hz, 2 H, ArH, NH), 4.47–4.58 (m, 2 H), 3.33–3.40 (m, 4 H), 2.88 (qd, *J* = 48.9, 16.2, 3.2 Hz, 2 H), 2.64 (t, *J* = 5.5 Hz, 1 H), 2.55–2.61 (m, 1 H), 2.30–2.33 (m, 1 H), 1.35 (s, 3 H, CH_3_), 1.22 (d, *J* = 9.2 Hz, 1 H), 1.11 (t, *J* = 6.9 Hz, 6 H, CH_3_), 0.68 (s, 3 H, CH_3_). ^13^C NMR (100 MHz, DMSO-*d*_6_, ppm) *δ*: 174.79, 159.97, 147.72, 141.20, 130.05, 128.02, 127.20, 126.31, 124.97, 110.87, 110.34, 49.76, 44.06, 40.08, 38.04, 29.90, 29.41, 25.56, 21.01, 12.44. HRMS-ESI: 427.2862 calcd for C_28_H_35_N_4_ [M+H]^+^, found: 427.2877.

*4-(4-(Diethylamino)phenyl)-7,7-dimethyl-N-(3-pyridylmethyl)-5,6,7,8-tetrahydro-6,8-methoxy quinazolin-2-amine* (**1n**): the synthetic method of **1i** was taken as a reference, **1f** and 3-chloromethylpyridine were selected as raw materials, a yellow solid was obtained with the yield of 50.2%, melting point: 139–141 °C. ^1^H NMR (400 MHz, DMSO-*d*_6_, ppm) *δ*: 8.50 (d, *J* = 4.8 Hz, 1 H, ArH), 7.72 (t, *J* = 8.0 Hz, 1 H, ArH), 7.62 (d, *J* = 8.4 Hz, 2 H, ArH), 7.33 (d, *J* = 7.9 Hz, 1 H, ArH), 7.19–7.24 (m, 2 H, ArH), 6.66 (d, *J* = 8.6 Hz, 2 H, ArH, NH), 4.56–4.68 (m, 2 H), 2.89 (q, *J* = 51.9, 15.5 Hz, 2 H), 2.64 (t, *J* = 5.6 Hz, 1 H), 2.56–2.61 (m, 1 H), 2.32 (s, 1 H), 1.35 (s, 3 H, CH_3_), 1.23 (d, *J* = 9.0 Hz, 1 H), 1.10 (t, *J* = 6.9 Hz, 6 H), 0.68 (s, 3 H, CH_3_). ^13^C NMR (100 MHz, DMSO-*d*_6_, ppm) *δ*: 174.92, 160.17, 159.88, 148.60, 147.74, 136.42, 130.06, 124.83, 121.68, 120.70 111.16, 110.33, 49.75, 46.15, 43.58, 38.04, 29.90, 29.40, 25.55, 21.00, 12.43. HRMS-ESI: 428.2814 calcd for C_27_H_34_N_5_ [M+H]^+^, found: 428.2809.

#### 4.1.4. Synthesis of Intermediate 3-(4-Methoxybenzylidene)-1, 7, 7-Trimethylbicyclo [2.2.1] Heptan-2-One (**2a**)

The synthetic method of **1a** was taken as a reference, camphor and 4-methoxybenzaldehyde were selected as raw materials, light yellow crystals were obtained with the yield of 56.8%, melting point: 97–98 °C. ^1^H NMR (400 MHz, DMSO-*d_6_*, ppm) *δ*: 7.45–7.48 (m, 2 H, ArH), 7.22 (s, 1 H, ArCH=C), 6.93–6.96 (m, 2 H, ArH), 3.86 (s, 3 H, OCH_3_), 3.11 (d, *J* = 4.4 Hz, 1 H), 2.18–2.22 (m, 1 H), 1.76–1.82 (m, 1 H), 1.53–1.65 (m, 2 H), 1.02–1.05 (m, 6 H, CH_3_), 0.83 (s, 3 H, CH_3_). ^13^C NMR (100 MHz, DMSO-*d_6_*, ppm) *δ*: 9.31, 18.40, 20.55, 25.92, 30.85, 46.79, 49.23, 55.33, 57.03, 114.18, 127.35, 128.30, 131.39, 140.07, 160.11, 208.27. HRMS-ESI: 271.1698 calcd for C_18_H_23_O_2_ [M+H]^+^, found: 271.1700.

#### 4.1.5. Synthesis of Camphoryl Pyrimidine Amines **2b**~**2f**

*4-(4-Methoxyphenyl)-8,9,9-trimethyl-5,6,7,8-tetrahydro-5,8-methylquinazolin-2-amine* (**2b**): the synthetic method of **1e** was taken as a reference, 2a was selected as raw material, a white solid was obtained with the yield of 30.8%, melting point: 183–184 °C. ^1^H NMR (400 MHz, DMSO-*d_6_*, ppm) *δ*: 0.56 (s, 3 H, CH_3_), 0.96 (s, 3 H, CH_3_), 1.15 (s, 3 H, CH_3_), 1.18–1.28 (m, 2 H), 1.84–1.90 (m, 1 H), 2.14–2.17 (m, 1 H), 3.05 (d, *J* = 3.6 Hz, 1 H), 3.81 (s, 3 H, OCH_3_), 6.26 (s, 2 H, NH_2_), 7.04 (d, *J* = 8.8 Hz, 2 H, ArH), 7.75 (d, *J* = 8.8 Hz, 2 H, ArH). ^13^C NMR (100 MHz, DMSO-*d_6_*, ppm) *δ*: 10.71, 19.22, 20.15, 26.28, 32.08, 49.83, 54.01, 55.49, 55.70, 114.28, 122.99, 129.89, 130.84, 155.06, 160.56, 162.54, 181.11. HRMS-ESI: 310.1919 calcd for C_19_H_24_N_3_O [M+H]^+^, found: 310.1920.

*N-(2-Methoxyethyl)-4-(4-methoxyphenyl)-8,9,9-trimethyl-5,6,7,8-tetrahydro-5,8-methyl Oxyquinazolin-2-amine* (**2c**): the synthetic method of **1i** was taken as a reference, **2a** and 2-bromoethyl methyl ether were selected as raw materials, a yellow oil was obtained with the yield of 73.0%. ^1^H NMR (400 MHz, DMSO-*d_6_*, ppm) *δ*: 0.56 (s, 3 H, CH_3_), 0.96 (s, 3 H, CH_3_), 1.15 (s, 3 H, CH_3_), 1.24 (m, 2 H), 1.88–2.02 (m, 1 H), 2.16–2.18 (m, 1 H), 3.08 (d, *J* = 3.6 Hz, 1 H), 3.31 (s, 3 H, COCH_3_), 3.49–3.50 (m, 4 H), 3.82 (s, 3 H, ArOCH_3_), 6.70 (s, 1 H, NH), 7.05–7.07 (m, 2 H, ArH), 7.80 (d, *J* = 8.8 Hz, 2 H, ArH). ^13^C NMR (100 MHz, DMSO-*d_6_*, ppm) *δ*: 10.04, 19.08, 20.01, 26.02, 31.93, 41.37, 49.97, 53.93, 55.32, 55.60, 58.69, 71.65, 113.76, 123.89, 129.79, 131.15, 154.95, 160.51, 161.08, 181.34. HRMS-ESI: 368.2338 calcd for C_22_H_30_N_3_O_2_ [M+H]^+^, found: 368.2397.

*N-(3-Fluorobenzyl)-4-(4-methoxyphenyl)-8,9,9-trimethyl-5,6,7,8-tetrahydro-5,8-methoxy quinazolin-2-amine* (**2d**): the synthetic method of **1i** was taken as a reference, **2a** and 1-bromomethyl-3-fluorobenzene were selected as raw materials, a gray solid was obtained with the yield of 39.1%, melting point: 93–96 °C. ^1^H NMR (400 MHz, DMSO-*d_6_*, ppm) *δ*: 0.54 (s, 3 H, CH_3_), 0.96 (s, 3 H, CH_3_), 1.16–1.27 (m, 5 H, CH_3_, CH_2_), 1.87–1.90 (m, 1 H), 2.16–2.17 (m, 1 H), 3.07–3.08 (m, 1 H), 3.81 (s, 3 H, OCH_3_), 4.49–4.52 (m, 2 H), 7.02–7.05 (m, 2 H, ArH, NH), 7.09–7.14 (m, 2 H, ArH), 7.39–7.43 (m, 3 H, ArH), 7.75–7.78 (m, 2 H, ArH), ^13^C NMR (100 MHz, DMSO-*d_6_*, ppm) *δ*: 10.64, 19.19, 20.19, 26.20, 32.06, 44.24, 49.85, 53.96, 55.45, 55.70, 114.34, 115.19 (d, *J* = 21.13 Hz), 123.04, 129.78, 129.86, 130.93, 137.72–137.75 (d, *J* = 3.0 Hz), 160.25, 160.44, 161.14–162.65 (d, *J* = 151.9 Hz), 181.20. HRMS-ESI: 418.2295 calcd for C_26_H_29_FN_3_O [M+H]^+^, found: 418.2296.

*N-Benzyl-4-(4-methoxyphenyl)-8,9,9-trimethyl-5,6,7,8-tetrahydro-5,8-methylquinazoline-2- Amine* (**2e**): the synthetic method of **1i** was taken as a reference, **2a** and benzyl chloride were selected as raw materials, a yellow solid was obtained with the yield of 44.2%, melting point: 114–115 °C. ^1^H NMR (400 MHz, DMSO-*d_6_*, ppm) *δ*: ^1^H NMR (400 MHz, DMSO-*d_6_*): 0.55 (s, 3 H, CH_3_), 0.96 (s, 3 H, CH_3_), 1.24–1.25 (m, 5 H, CH_3_, CH_2_), 1.85–1.90 (m, 1 H), 2.14–2.18 (m, 1 H), 3.07–3.08 (m, 1 H), 3.81 (s, 3 H, OCH_3_), 4.50–4.54 (m, 2 H), 7.02–7.05 (m, 2 H, ArH, NH), 7.18–7.19 (m, 1 H, ArH), 7.21–7.31 (m, 2 H, ArH), 7.38–7.39 (m, 3 H, ArH), 7.75–7.79 (m, 2 H, ArH). ^13^C NMR (100 MHz, DMSO-*d_6_*, ppm) *δ*: 10.66, 19.20, 20.20, 49.86, 53.96, 55.70, 114.33, 122.95, 126.84, 127.96, 128.51, 129.86, 141.60, 160.63 (d, *J* = 39.24 Hz). HRMS-ESI: 400.2389 calcd for C_26_H_30_N_3_O [M+H]^+^, found: 400.2395.

*N-(2,4-Difluorobenzyl)-4-(4-methoxyphenyl)-8,9,9-trimethyl-5,6,7,8-tetrahydro-5,8- Methylquinazolin-2-amine* (**2f**): the synthetic method of **1i** was taken as a reference, **2a** and 2,4-difluorobenzyl bromide were selected as raw materials, a gray solid was obtained with the yield of 43.3%, melting point: 59–61 °C. ^1^H NMR (400 MHz, DMSO-*d_6_*, ppm) *δ*: 0.54 (s, 3 H, CH_3_), 0.96 (s, 3 H, CH_3_), 1.16–1.26 (m, 5 H, CH_3_, CH_2_), 1.87–1.88 (m, 1 H), 2.16–2.17 (m, 1 H), 3.07–3.08 (m, 1 H), 3.81 (s, 3 H, OCH_3_), 4.52–4.55 (m, 2 H), 7.02–7.05 (m, 3 H, ArH, NH), 7.15–7.20 (m, 1 H, ArH), 7.44–7.50 (m, 2 H, ArH), 7.74–7.77 (m, 2 H, ArH). ^13^C NMR (100 MHz, DMSO-*d_6_*, ppm) *δ*: 10.02, 19.05, 19.99, 25.91, 31.88, 39.07 (d, *J* = 4.0 Hz), 49.97, 54.06, 55.35, 55.74, 103.47 (t, *J* = 25.0 Hz), 110.88 (dd, *J* = 4.0, 17.1 Hz), 113.83, 122.85 (d, *J* = 4.0 Hz), 124.29, 129.83, 131.07 (dd, *J* = 6.0, 7.0 Hz), 159.90, 160.55, 160.66, 161.09 (d, *J* = 259.8 Hz), 162.04 (d, *J* = 247.5 Hz), 181.73. HRMS-ESI: 436.2200 calcd for C_26_H_28_F_2_N_3_O [M+H]^+^, found: 436.2203.

### 4.2. Antibacterial Activities Assay

*K. pneumoniae*, *S. pneumoniae*, *P. aeruginosa*, *S. aureus*, *Escherichia coli* (*E. coli*), methicillin-resistant Staphylococcus aureus (MRSA), Bacillus cereus (*B. cereus*), and Candida albicans (*C. albicans*) were selected as test bacterial and fungi strains, which were provided by the Laboratory of the Third People’s Hospital of Yancheng City. Ketoconazole and amikacin were selected as the control drugs for inhibiting fungi and bacteria; the antibacterial activities of the synthesized pinanyl pyrimidine amine derivatives and camphoryl pyrimidine amine derivatives were evaluated by the micro-dilution method. A total of 100 μL of purified water was added into 96-well plate from the 2nd to the 12th well firstly; then, the test compounds **1e**~**1n** and **2b**~**2f** solutions, the positive control drug ketoconazole and amikacin solutions at the concentration of 100 μg/mL with methanol were added to the first well in the volume of 100 μL. The test compounds and positive control drugs were diluted twice on 96-well plates, and a series of concentration gradients (1024–1.0 μg/mL) were prepared from the first to the 12th well. Pure DMSO as a reference, 100 μL of pre-prepared bacterial suspension, was added to achieve the required final concentration in a volume of 200 μL, and the solutions were mixed well. Finally, the 96-well plate was kept under 5% CO_2_ at 37 °C, the bacteria were cultured for 24 h, and the fungi were cultured for 48 h. The lowest concentration without turbidity was taken as the minimum inhibitory concentration of the sample against the test bacteria. Each sample was repeated three times for each test bacteria, the experimental data were recorded, and the results were averaged.

### 4.3. Anti-Inflammatory Activities Assay

The MTT method was used to detect the cell survival rate. Cells were evenly seeded into 96-well tissue culture plate at a density of 5×10^4^ cells per well and incubated for 12 h. Attachment of cells was controlled under the microscope. 10 mmol/L test compounds solution was diluted into 50 μM, 25 μM, 12.5 μM, 6.25 μM, 3.125 μM, 1.56 μM, 0.8 μM with serum-free medium. The cell supernatant was discarded, and the drug-containing solution was added into the 96-well plate as the required final concentration in a volume of 200 μL, the same volume of medium was added to both the positive control group and the negative control group, and the incubation was continued for 12 h. The cell supernatant was discarded, 5 μg/mL LPS serum-free medium was added, after an incubation period of 24 h, the MTT reagent was added (20 μL of a 5 mg/mL solution) to each well. Plates were further incubated for 1 h and then the assay was terminated by removing supernatant. Viability of cells was determined spectrophotometrically by measuring absorbance at 492 nm and background correction at 710 nm using a BMG POLARstar microplate reader; the data obtained were used to calculate the cell viability using SPSS software.

### 4.4. Molecular Docking

The complex crystal of tumor necrosis factor (TNF) with trifluorotolyl indole (PDB ID: 2AZ5) was chosen as the template to elucidate the binding mode of **1f** and TNF. Protein structure was downloaded from the PDB database (http://www.rcsb.org (accessed on 12th August 2022)) and saved in PDB file format. Pymol and Auto Dock software were used for molecular docking. The TNF kinase was defined as a receptor after the preparation of adding hydrogen atoms and deleting waters, adding charge and force fields, and the protein structure was optimized. The active cavity was defined as a grid box module: a grid box centered at (19.112, 7.014, 9.290) with a size of 18 × 18 × 18, the spaces of grid points were 1 Å. The images of binding mode were prepared using Auto Dock Vina software.

## Figures and Tables

**Table 1:** MIC (μg/mL) values of pinanyl pyrimidine amine and camphoryl pyrimidine amine derivatives.

Compd.	R^1^	R^2^	Bacterial							Fungi
			*K. pneumoniae*	*S. pneumoniae*	*P. aeruginosa*	*S. aureus*	*E. coli*	*MRSA*	*B. cereus*	*C. albicans*
**1e**	OH	H	32	32	16	32	8	64	8	>1024
**1f**	Cl	H	32	8	16	32	8	8	8	>1024
**1g**	OCH_3_	H	64	32	64	1	8	8	32	>1024
**1h**	N(C_2_H_5_)_2_	H	256	>1024	128	16	128	8	>1024	>1024
**1i**	OCH_3_	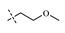	128	1	32	>1024	1	64	64	32
**1j**	OCH_3_	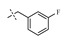	256	64	128	512	64	64	>1024	32
**1k**	OCH_3_	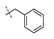	512	>1024	128	512	128	64	>1024	64
**1l**	OCH_3_	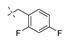	>1024	>1024	128	16	512	8	>1024	16
**1m**	N(C_2_H_5_)_2_	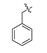	>1024	32	128	512	64	64	>1024	>1024
**1n**	N(C_2_H_5_)_2_	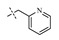	>1024	>1024	>1024	>1024	>1024	>1024	>1024	>1024
**2b**	OCH_3_	H	>1024	32	16	>1024	8	64	32	>1024
**2c**	OCH_3_	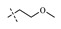	64	>1024	256	16	32	128	>1024	64
**2d**	OCH_3_	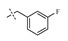	>1024	>1024	128	256	64	64	>1024	64
**2e**	OCH_3_	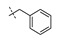	>1024	>1024	128	512	128	32	>1024	128
**2f**	OCH_3_	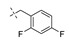	32	16	16	32	8	8	16	32
**amikacin ^b^**	-	-	32	2	32	1	2	1	4	-^a^
**ketoconazole ^b^**	-	-	-	-	-	-	-	-	-	16

^a^ “-”. No detection. ^b^. Amikacin is positive control against bacterium; ketoconazole is positive control against fungus.

**Table 2:** Properties of the target compounds and the control drugs.

Compd.	MW ^a^	CLogP ^b^	pKa ^c^	tPSA ^d^
**1e**	281	3.73	8.81	70.97
**1f**	300	4.86	-	50.74
**1g**	295	4.18	-	59.97
**1i**	353	5.01	-	55.21
**2f**	435	7.26	-	45.98
**amikacin**	585	−4.12	13.32	331.94
**1l**	421	6.74	-	45.98
**ketoconazole**	531	3.64	-	66.84

^a^: molecule weight (MW) was calculated from ChemBioDraw 20.0 and the digitals were rounded. ^b^: CLogP was predicted by ChemBioDraw 20.0. ^c^: pKa was predicted by ChemBioDraw 20.0, “-“: No data available. ^d^: tPSA (Topological polar surface area) was predicted by ChemBioDraw 20.0.

**Table 3:** Anti-inflammatory activity of compounds on RAW.

Compd.	(Anti-Inflammatory Activity)/μmol·L^−1^ (IC_50_ ± SD)
**1e**	2.32 ± 0.9
**1f**	1.37 ± 0.41
**1g**	3.0 ± 0.71
**1i**	4.86 ± 0.84
**2f**	1.87 ± 0.38
**Aspirin**	1.91 ± 0.35

## Data Availability

Not applicable.

## Supplementary Materials

The following are available online at www.mdpi.com/1420-3049/27/22/8104/s1, The ^1^H-NMR, ^13^C-NMR and HRMS spectra of the target compounds.
