# Supplementary material for: Synthesis and Biological Evaluation of Novel Pyrimidine Amine Derivatives Bearing Bicyclic Monoterpene Moieties
Source: Molecules. 2022 Nov 21;27(22):8104. doi: 10.3390/molecules27228104 (PMC9695817; doi:10.3390/molecules27228104)
Supplement: Supplementary file 1 [file molecules-27-08104-s001.zip › molecules-1996805-supplementary.pdf]

Synthesis and biological evaluation of novel pyrimidine amine derivatives bearing bicyclic monoterpene moieties

Mingguang Zhang [1], Yunyun Wang [2], Shifa Wang [3], Hongyan Wu [1,*]

(1 School of Pharmacology, Jiangsu Vocational College of Medicine, Yancheng 224005, China)
(2 School of Pharmacy and Jiangsu Province Key Laboratory for Inflammation and Molecular Drug Target, Nantong University, Nantong 226001, China)
(3 College of Chemical Engineering, Nanjing Forestry University, Nanjing 210037, China)
* Correspondence: why20133055@163.com (H.W.)

Spectral Copies of HRMS, ^{1}H, ^{13}C NMR Data Obtained in this Study

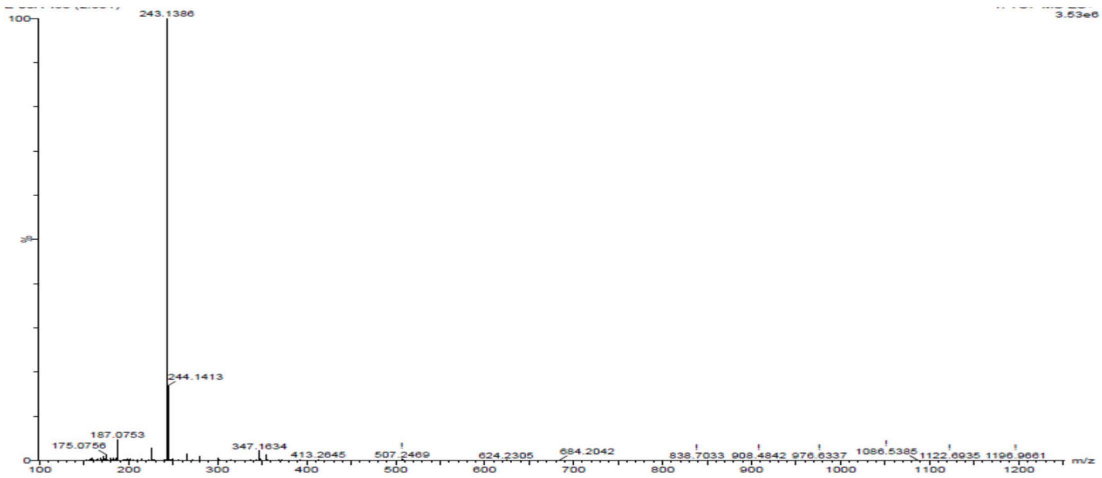

*Figure S1. HRMS spectrum of compound **1a***

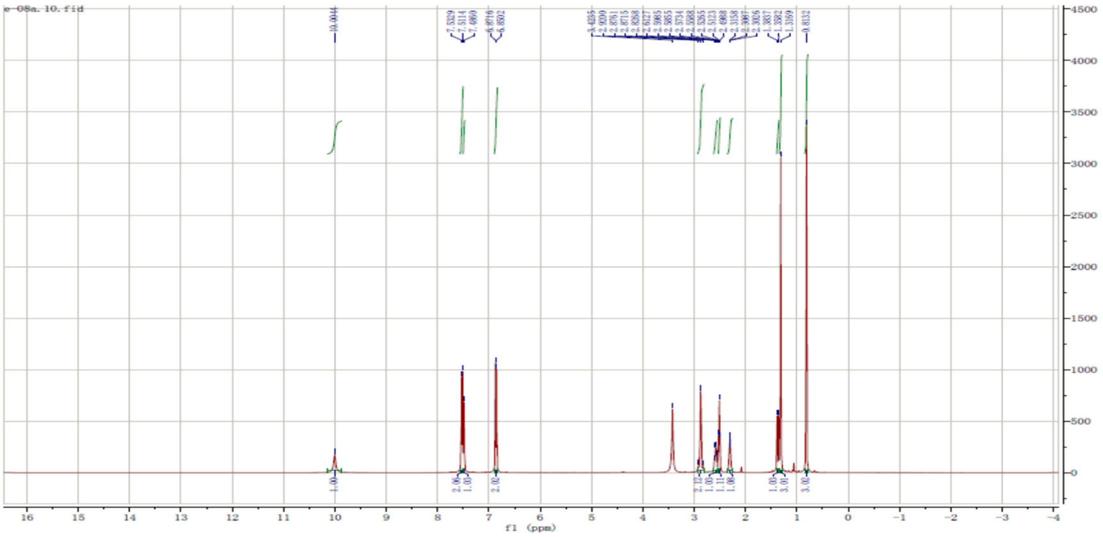

*Figure S2. ^{1}HNMR spectrum of compound **1a***

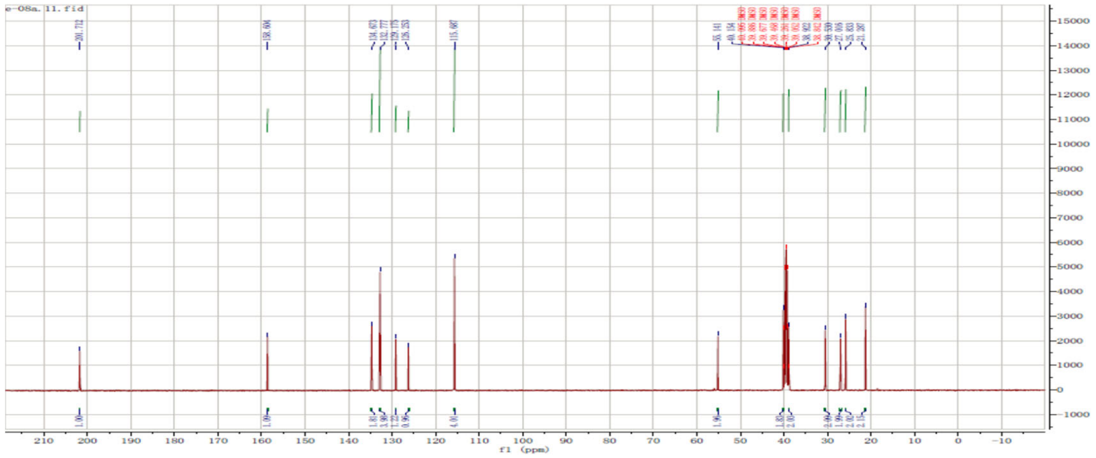

Figure S3. ^{13}C NMR spectrum of compound ***1a***

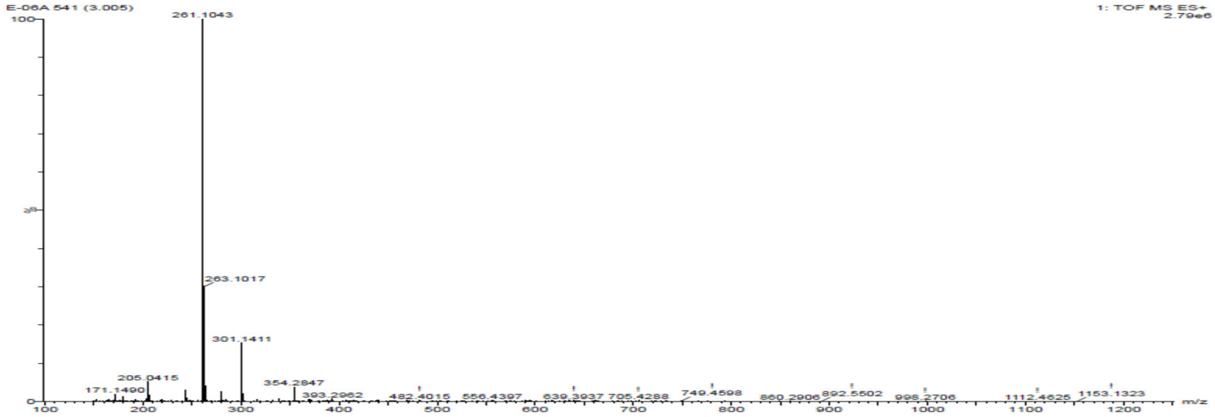

Figure S4. HRMS spectrum of compound ***1b***

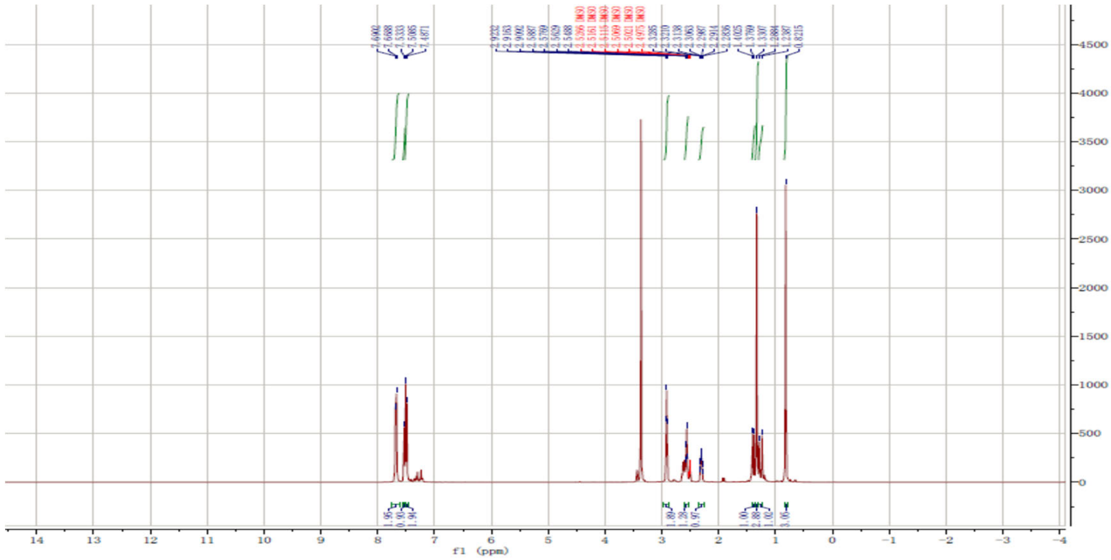

Figure S5. ^{1}HNMR spectrum of compound ***1b***

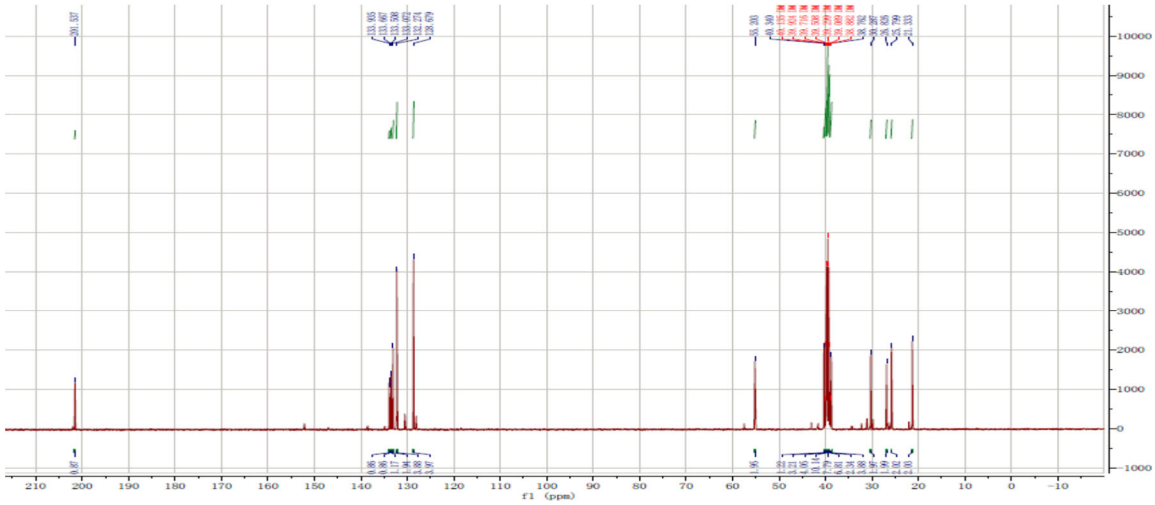

Figure S6. ^{13}*C NMR spectrum of compound* ***1b***

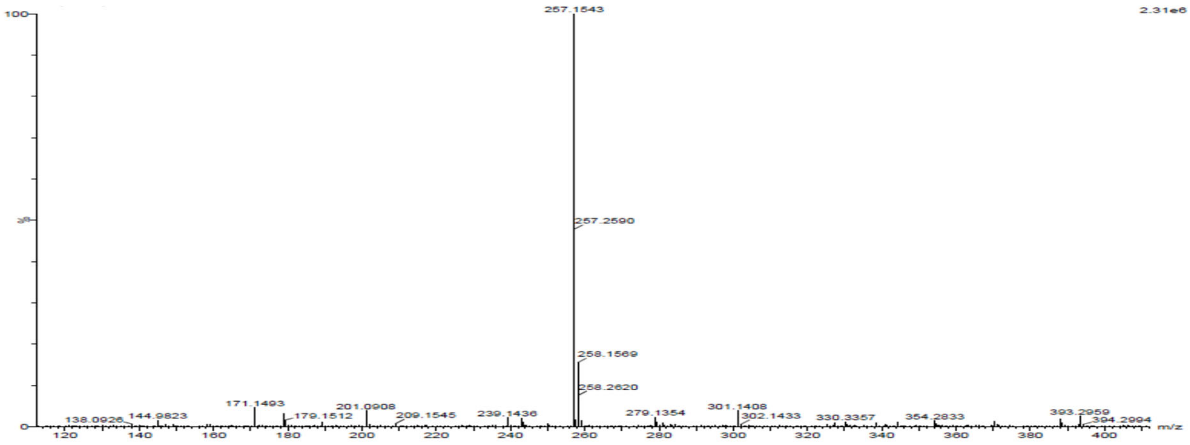

Figure S7. HRMS spectrum of compound ***1c***

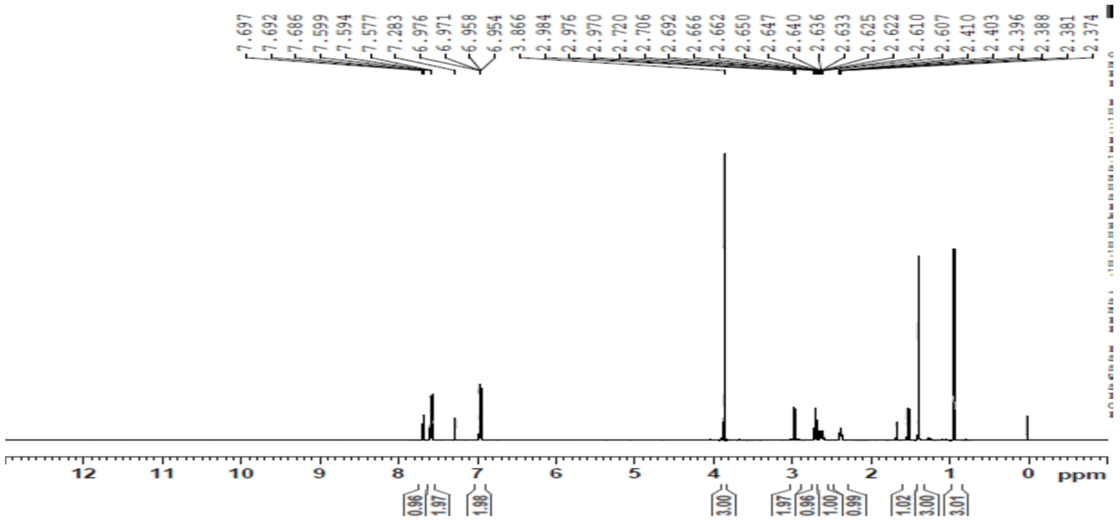

Figure S8. ^{1}*HNMR spectrum of compound* ***1c***

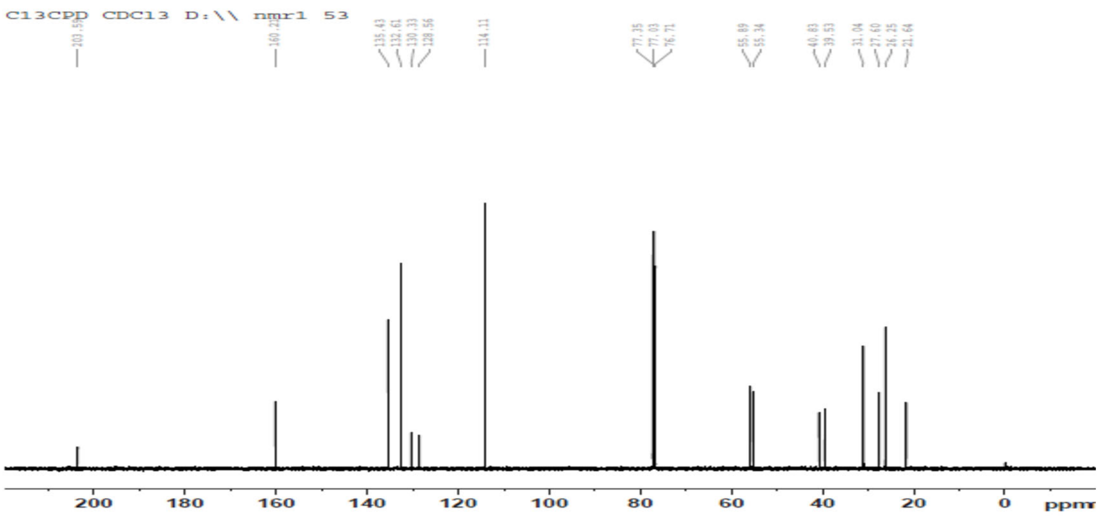

Figure S9. ^{13}*C NMR spectrum of compound* ***1c***

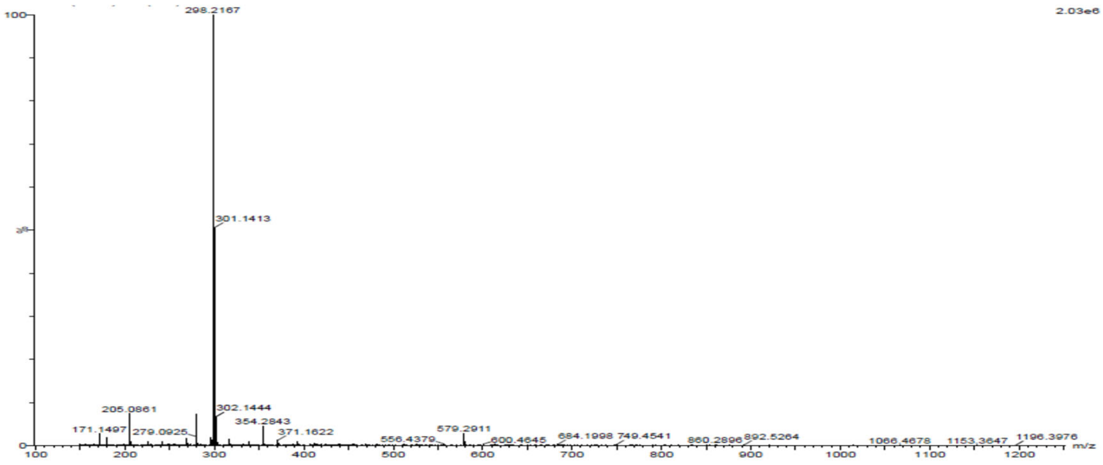

Figure S10. HRMS spectrum of compound ***1d***

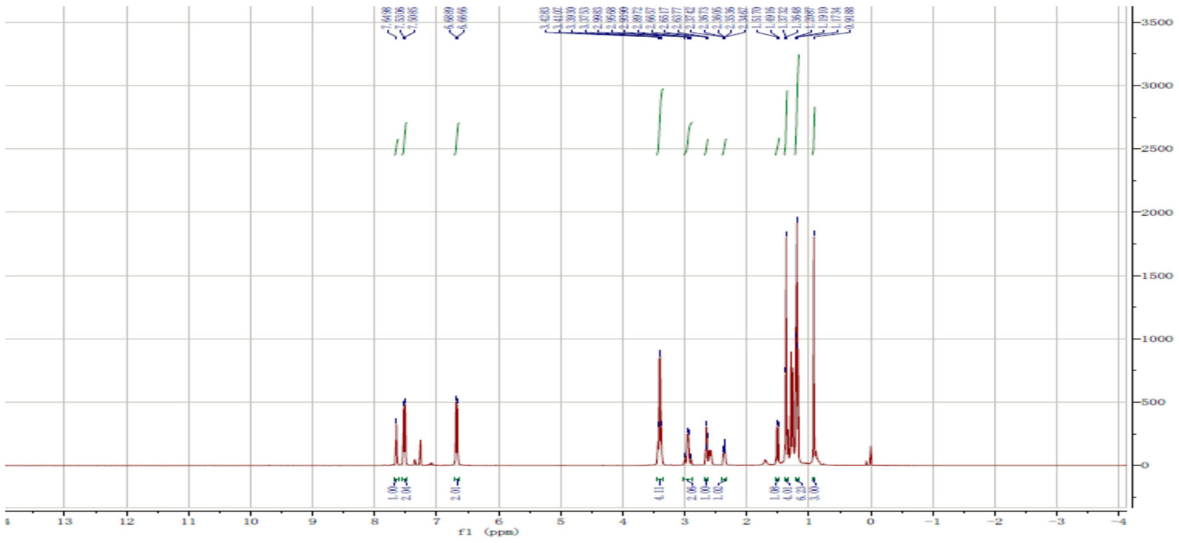

Figure S11. ^{1}*HNMR spectrum of compound* ***1d***

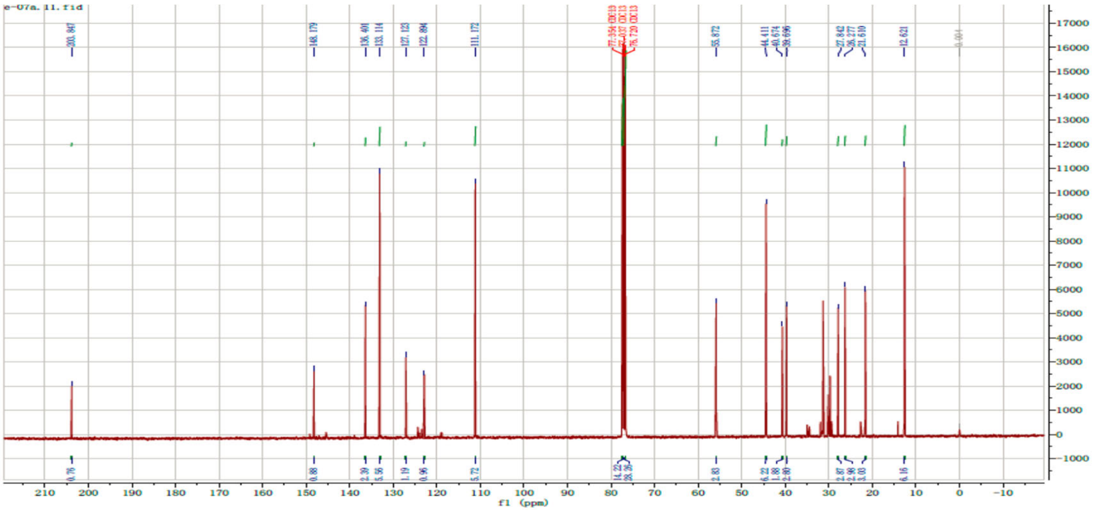

Figure S12. ^{13}C NMR spectrum of compound **1d**

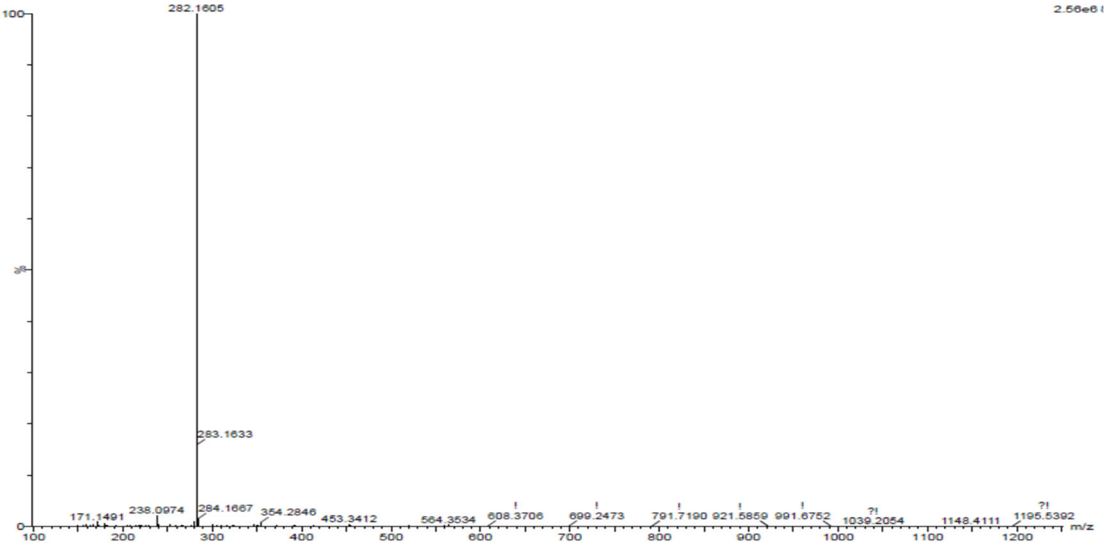

Figure S13. HRMS spectrum of compound **1e**

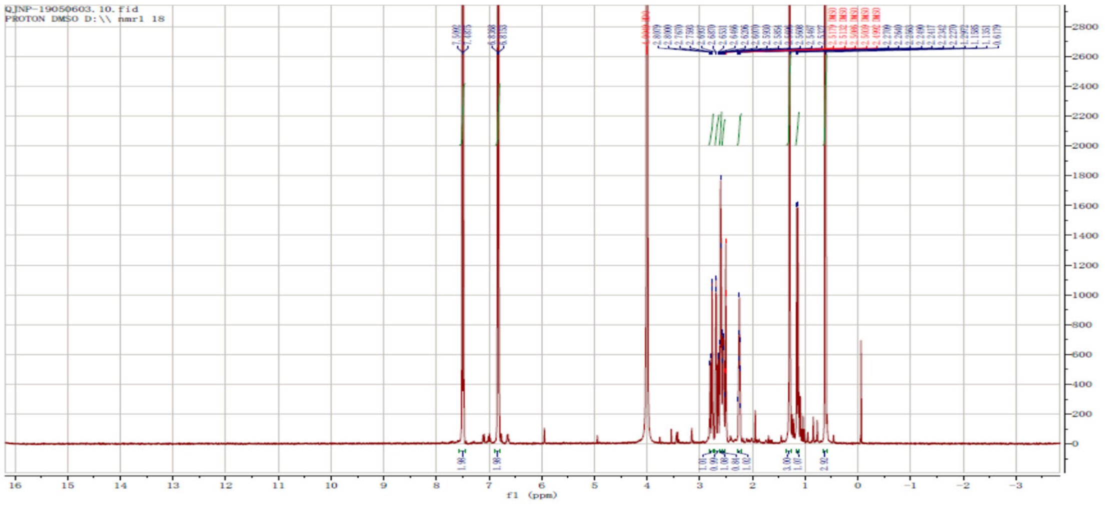

Figure S14. ^{1}HNMR spectrum of compound **1e**

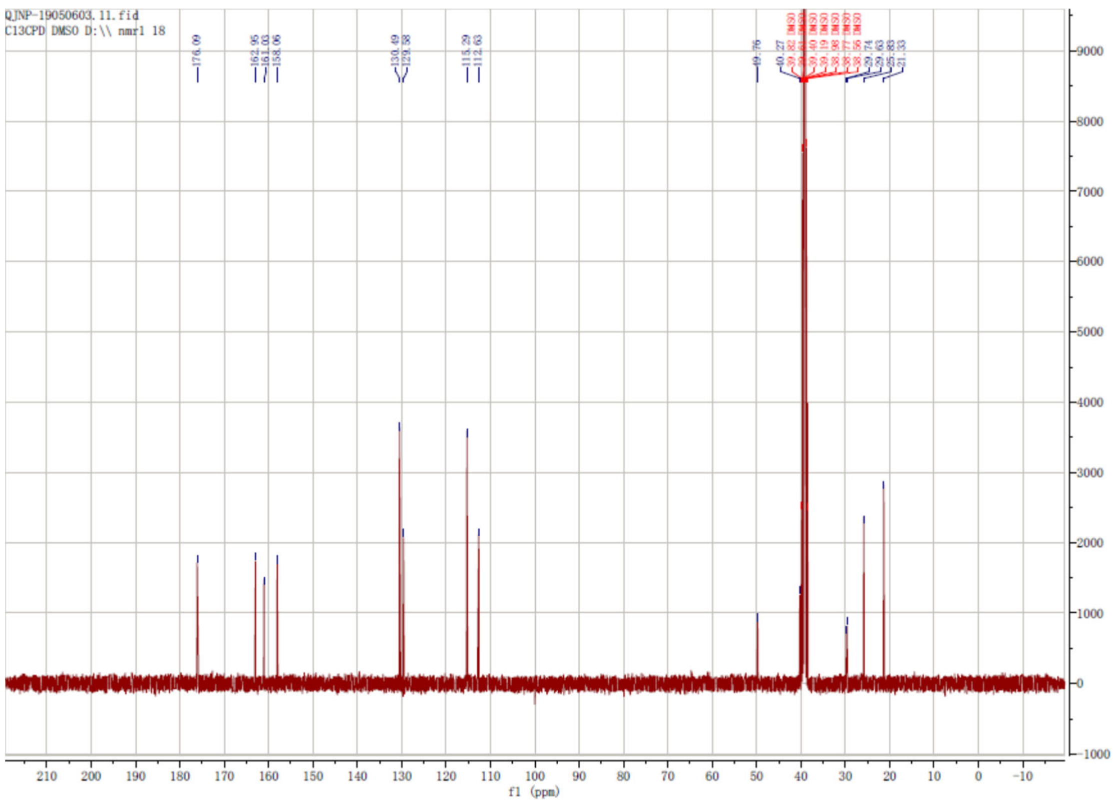

Figure S15. ^{13}C NMR spectrum of compound ***1e***

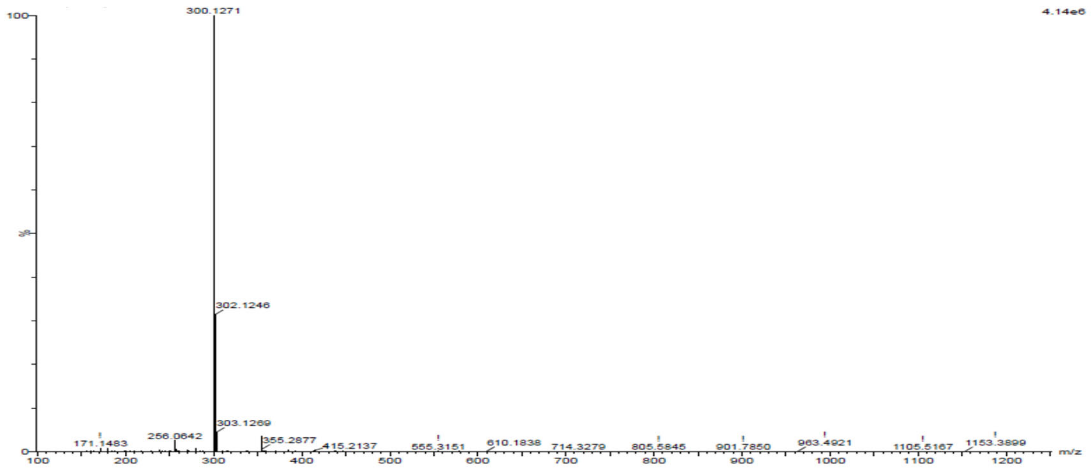

Figure S16. HRMS spectrum of compound ***1f***

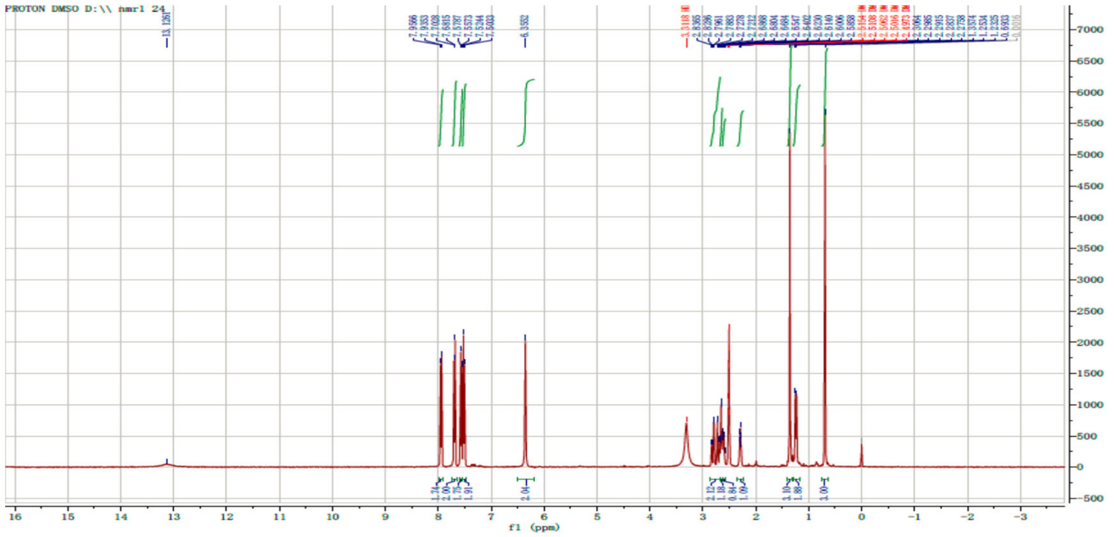

Figure S17. ^{1}HNMR spectrum of compound **1f**

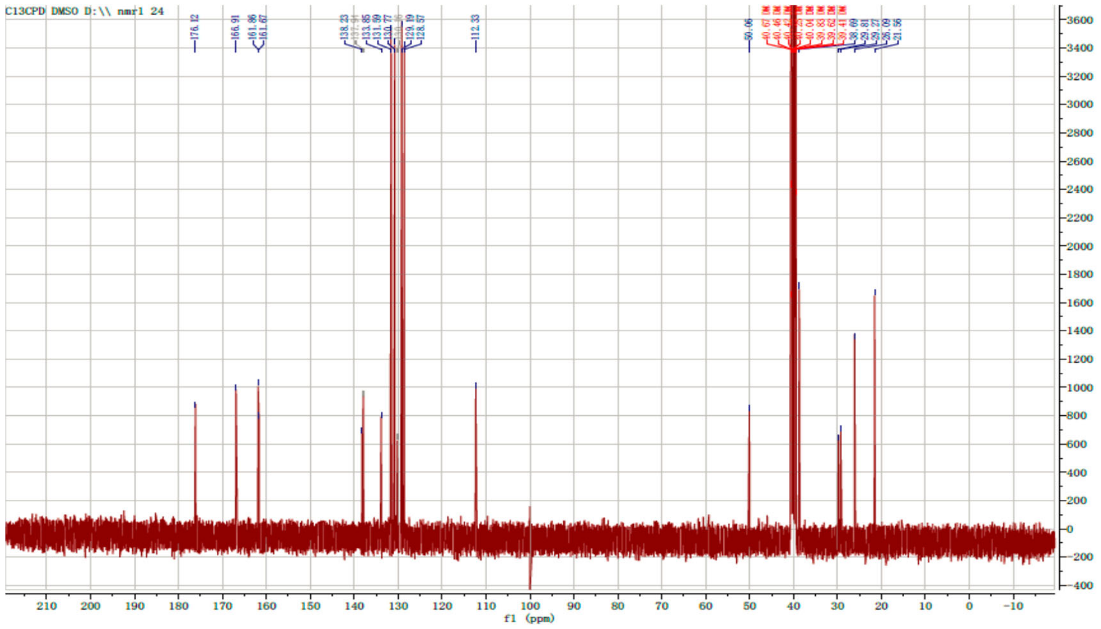

Figure S18. ^{13}C NMR spectrum of compound **1f**

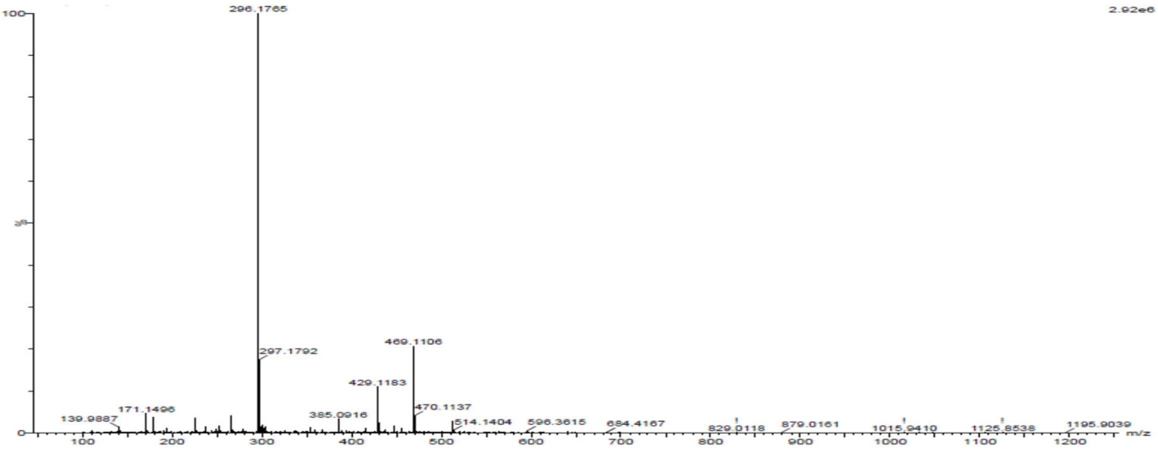

Figure S19. HRMS spectrum of compound **1g**

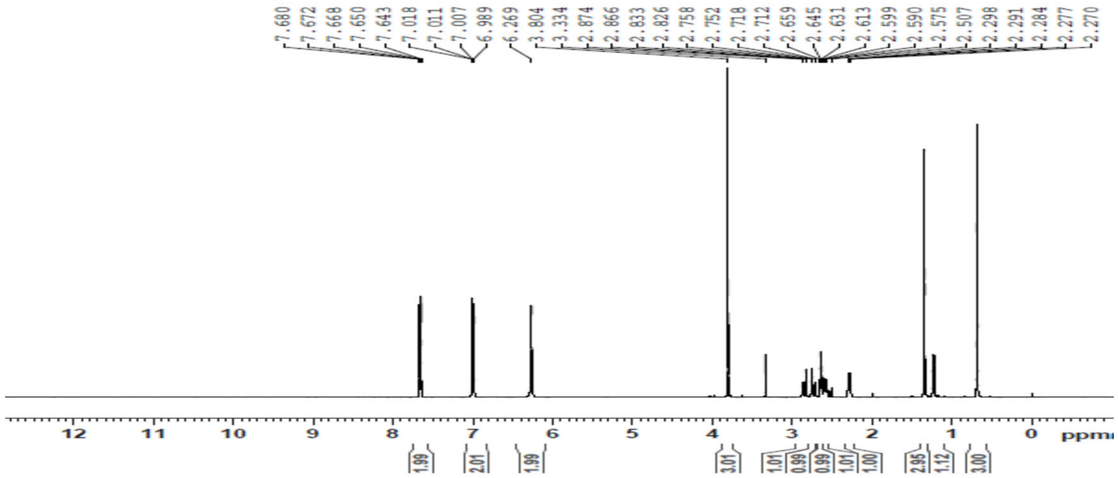

Figure S20. [1]*HNMR spectrum of compound* ***1g***

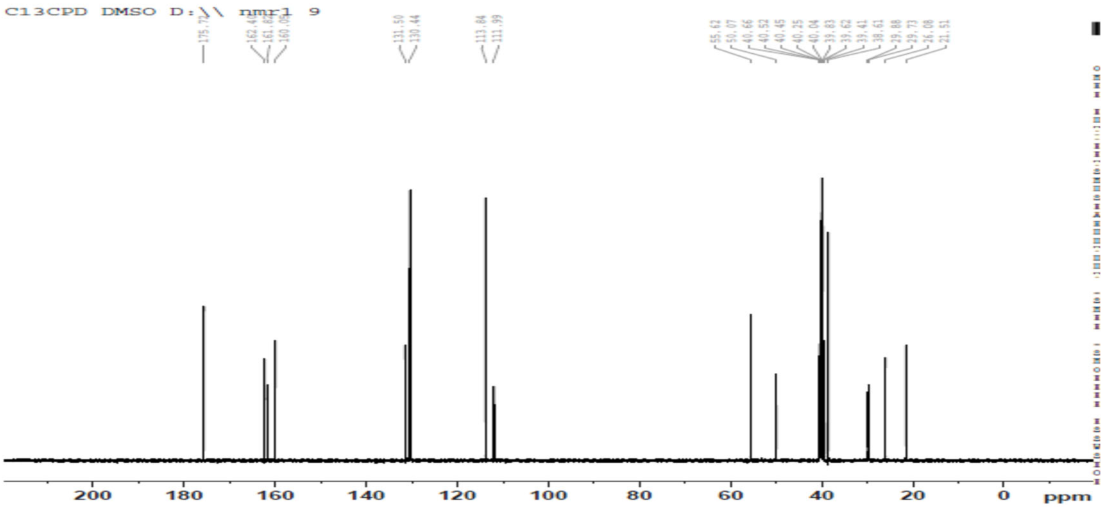

Figure S21. [13]*C NMR spectrum of compound* ***1g***

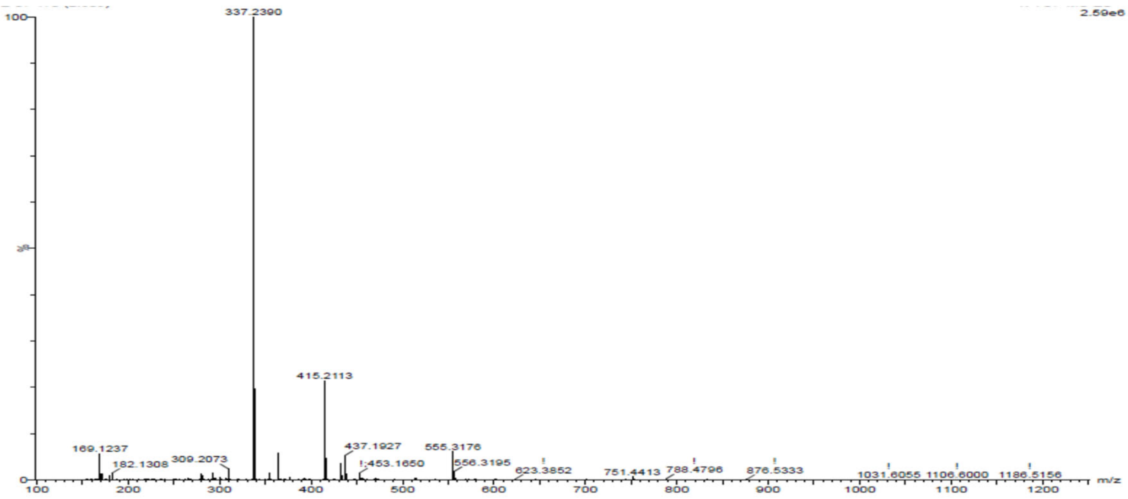

Figure S22. HRMS spectrum of compound ***1h***

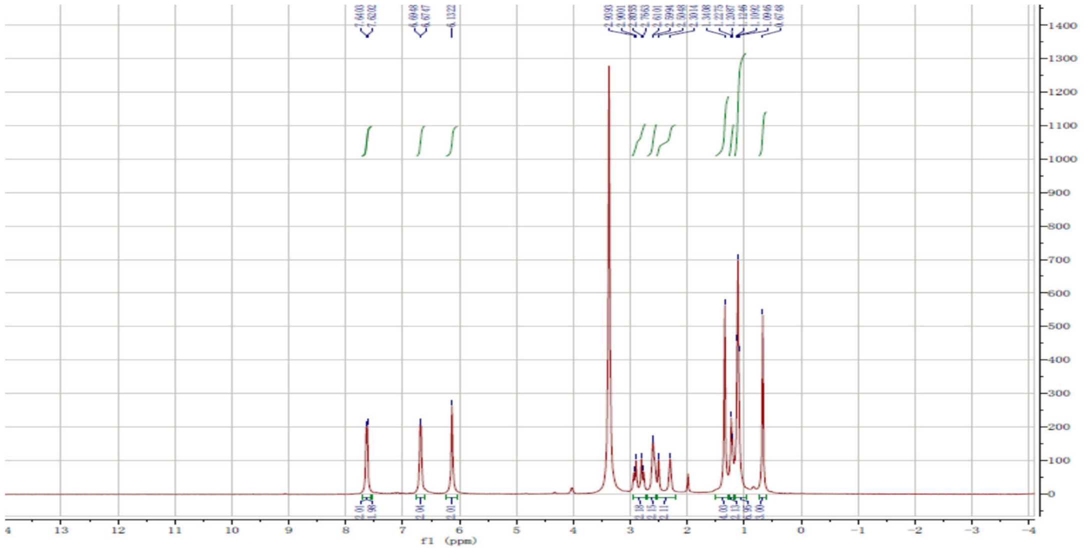

*Figure S23. ^{1}HNMR spectrum of compound **1h***

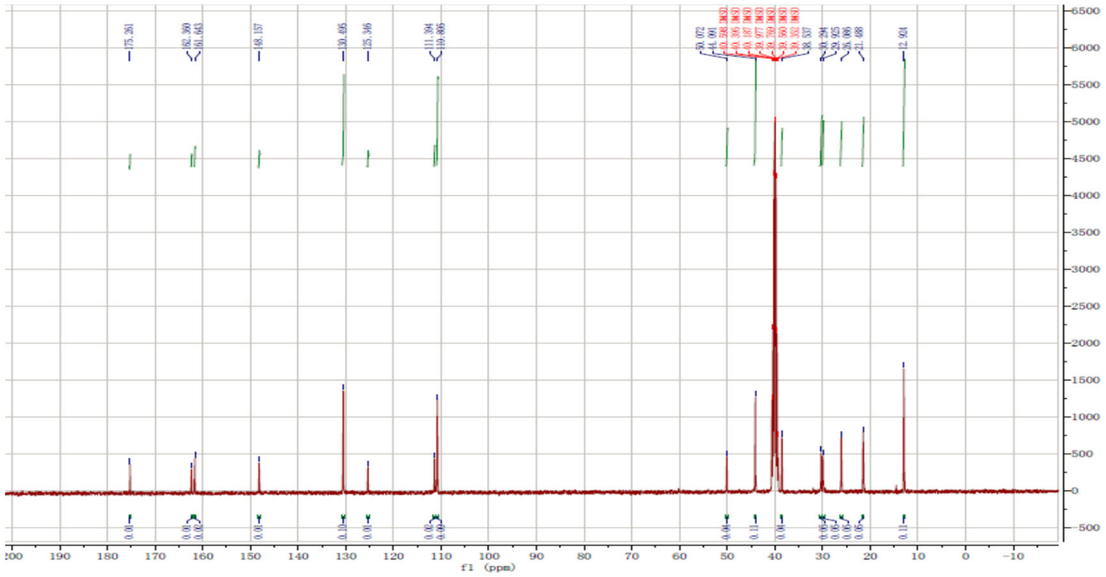

*Figure S24. ^{13}C NMR spectrum of compound **1h***

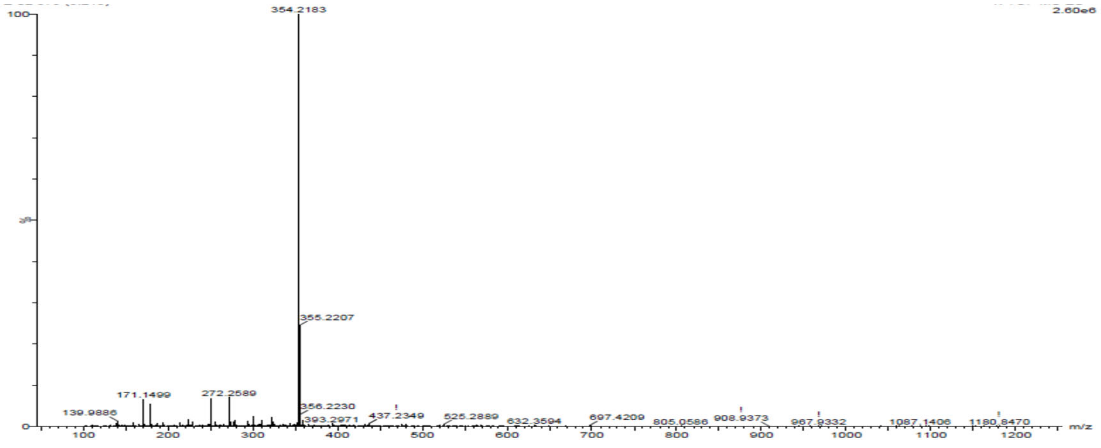

*Figure S25. HRMS spectrum of compound **1i***

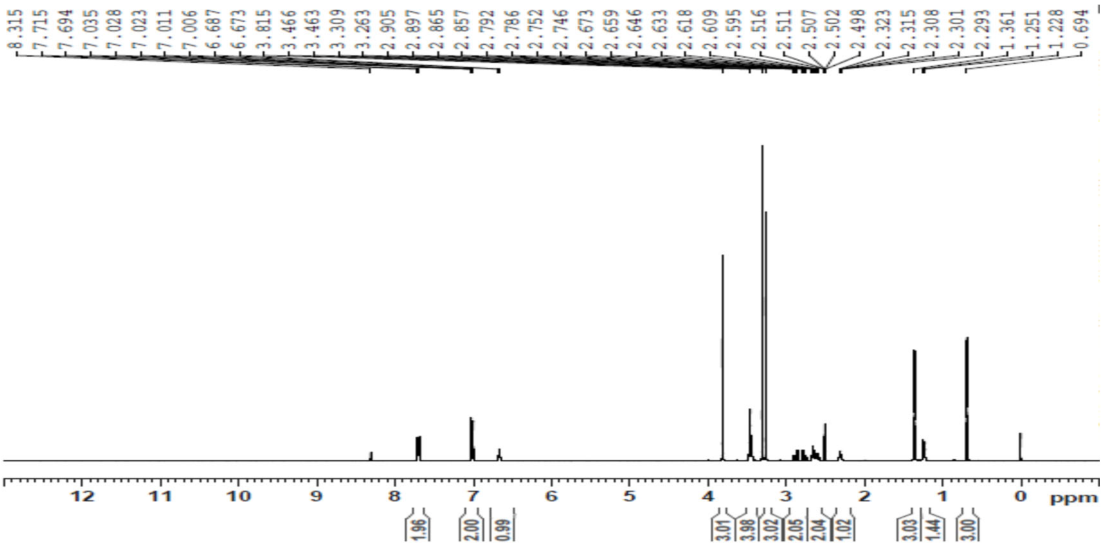

*Figure S26. ^{1}HNMR spectrum of compound **1i***

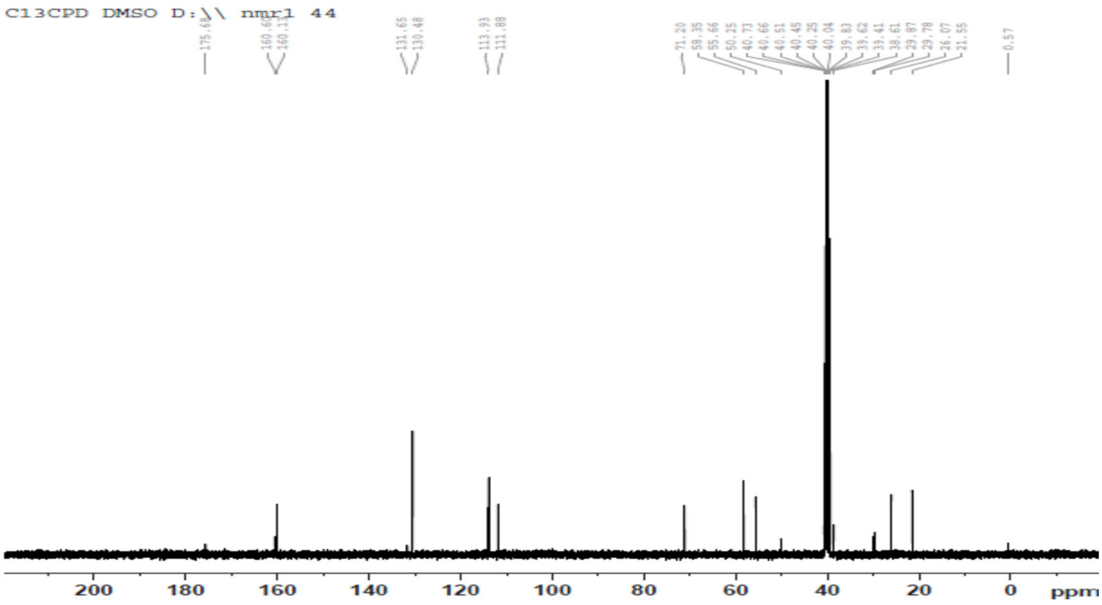

*Figure S27. ^{13}C NMR spectrum of compound **1i***

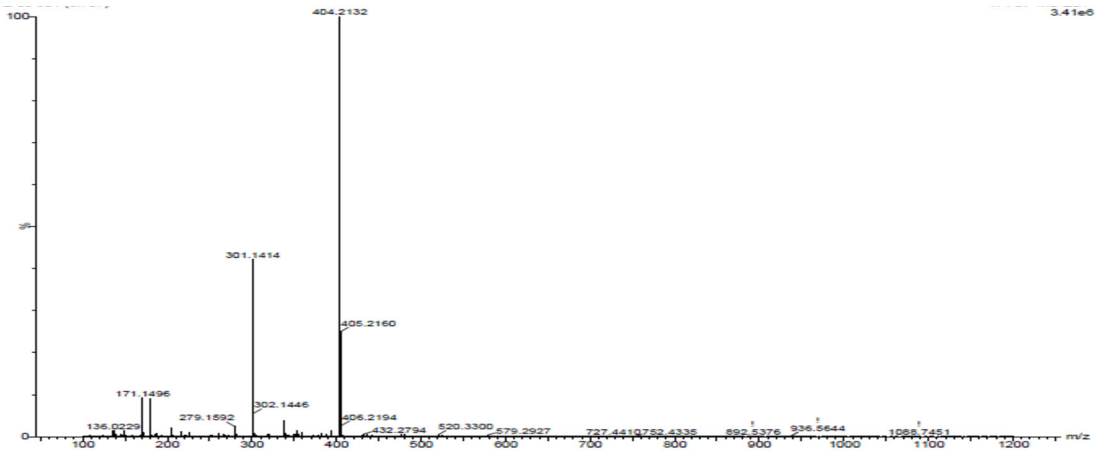

Figure S28. HRMS spectrum of compound **1j**

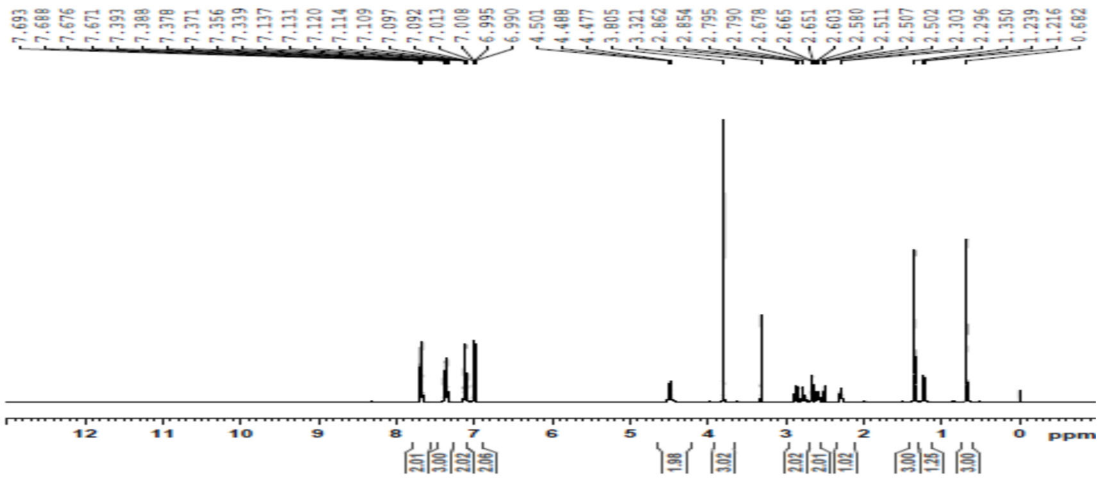

Figure S29. ^{1}HNMR spectrum of compound **1j**

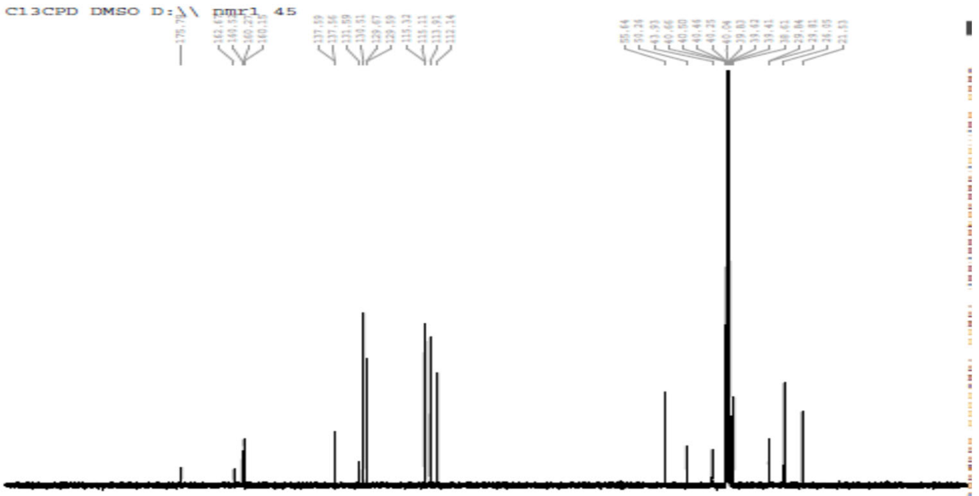

Figure S30. ^{13}C NMR spectrum of compound **1j**

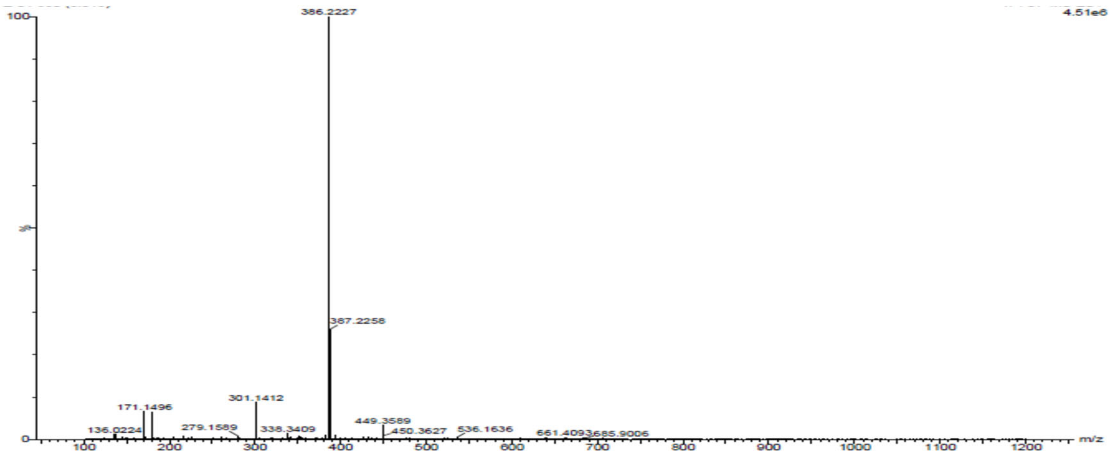

Figure S31. HRMS spectrum of compound ***1k***

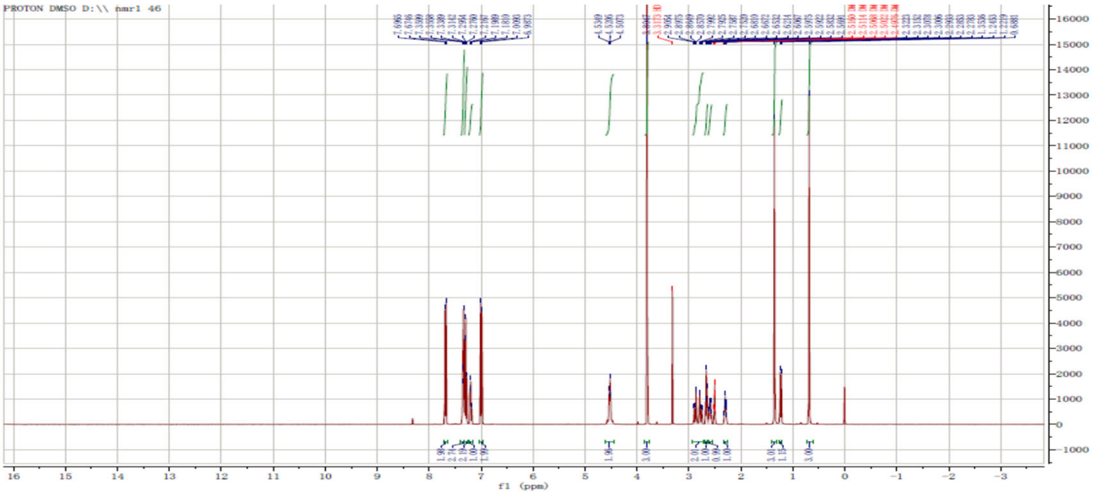

Figure S32. ^{1}HNMR spectrum of compound ***1k***

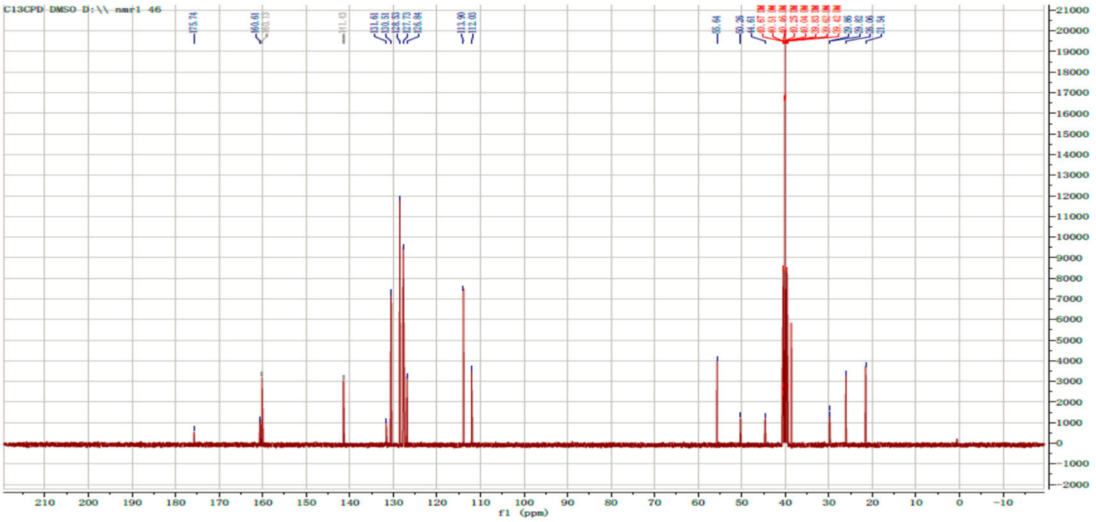

Figure S33. ^{13}C NMR spectrum of compound ***1k***

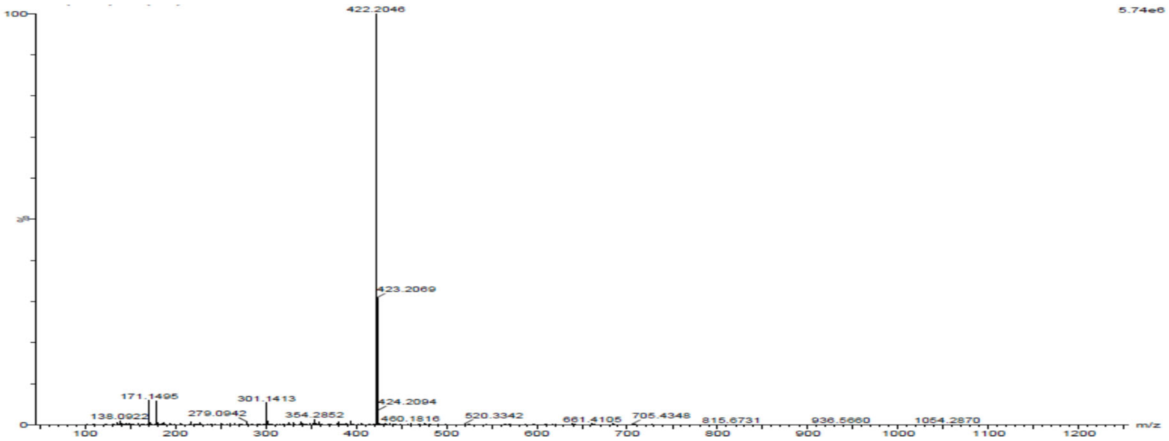

*Figure S34. HRMS spectrum of compound **1l***

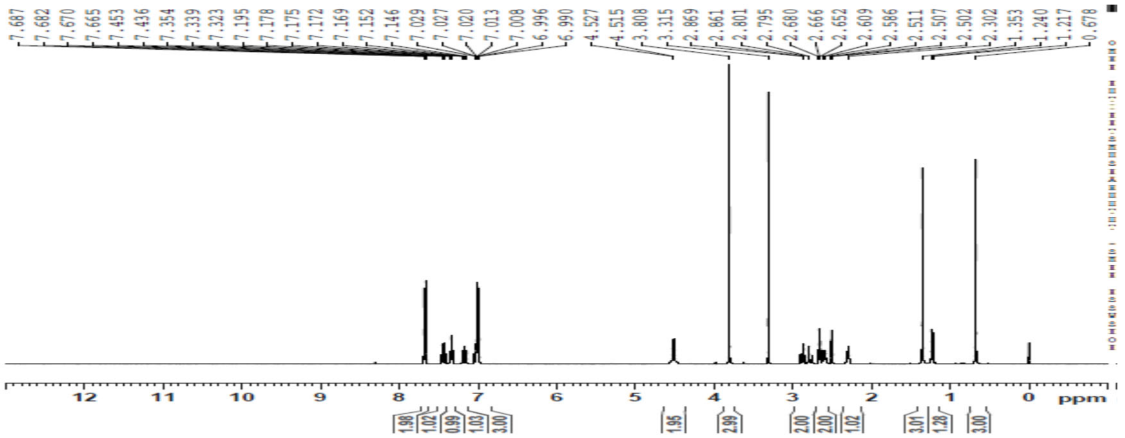

*Figure S35. ^{1}HNMR spectrum of compound **1l***

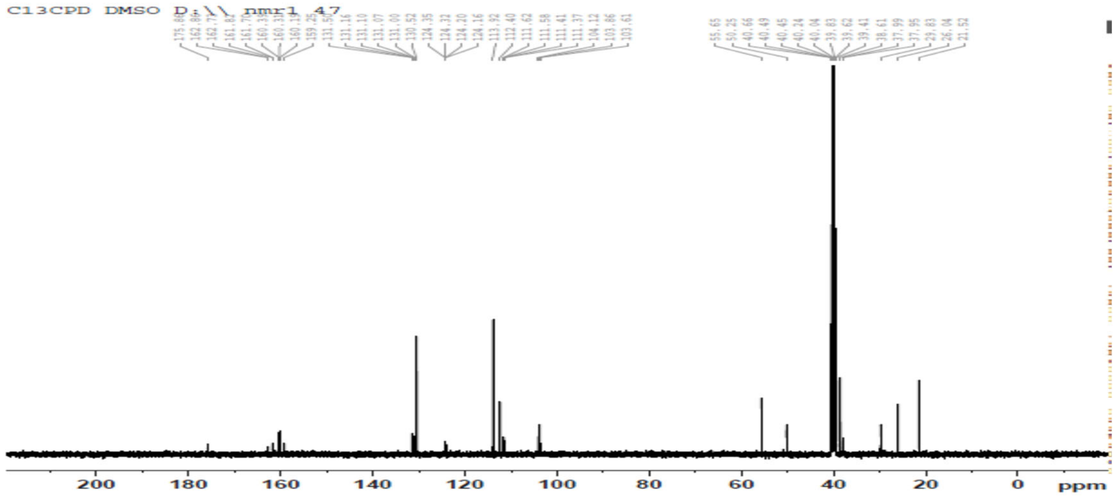

*Figure S36. ^{13}C NMR spectrum of compound **1l***

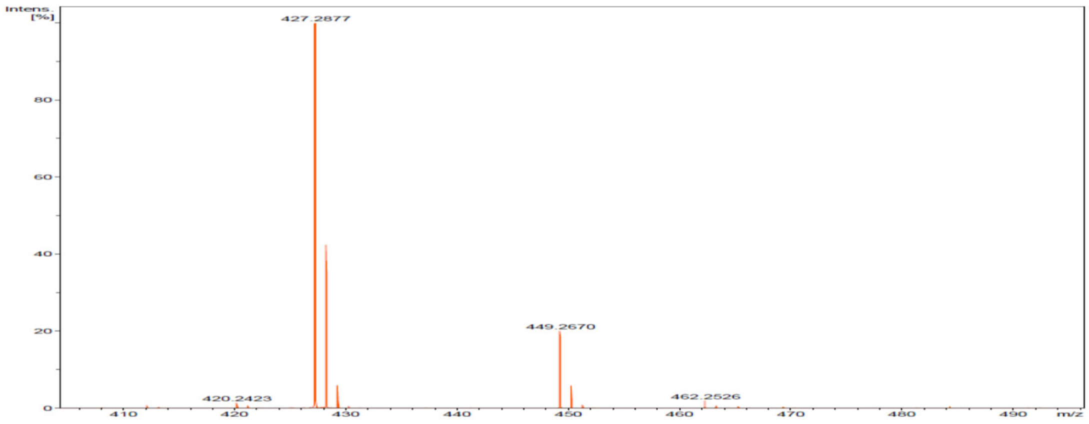

Figure S37. HRMS spectrum of compound ***1m***

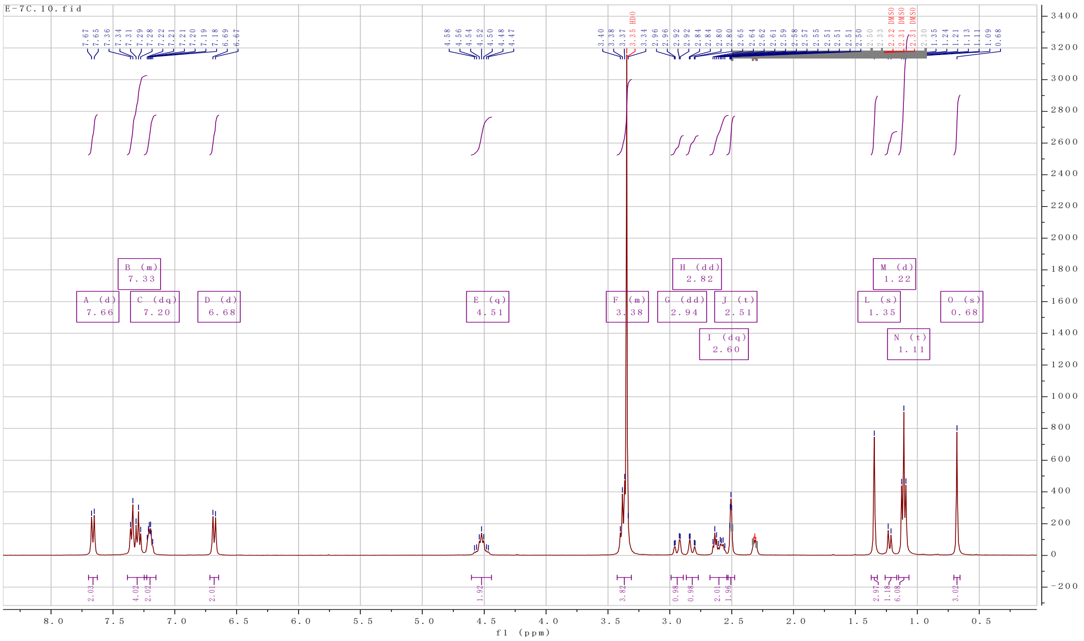

Figure S38. ^{1}HNMR spectrum of compound ***1m***

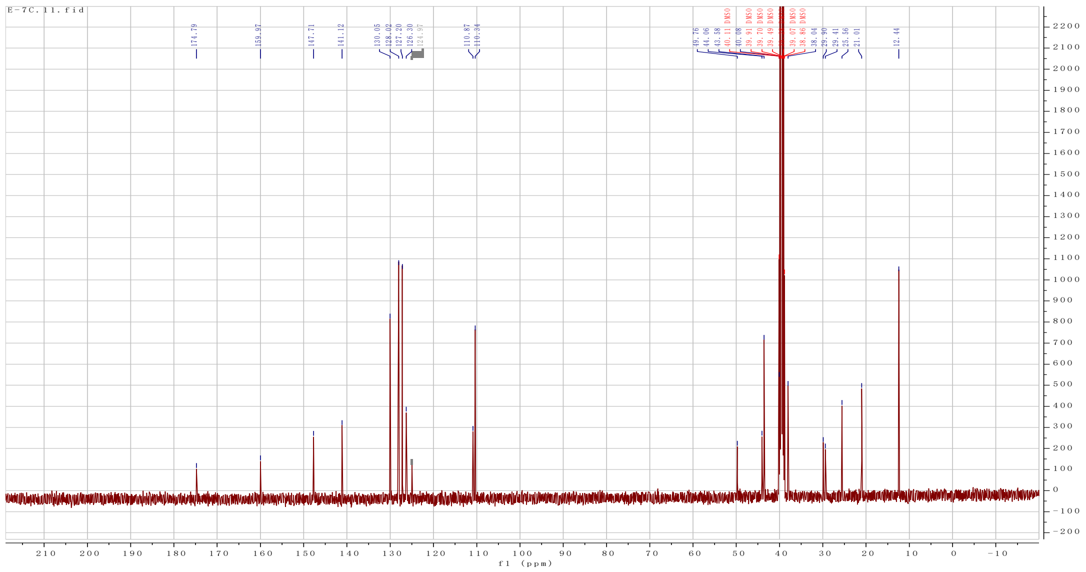

Figure S39. ^{13}C NMR spectrum of compound ***1m***

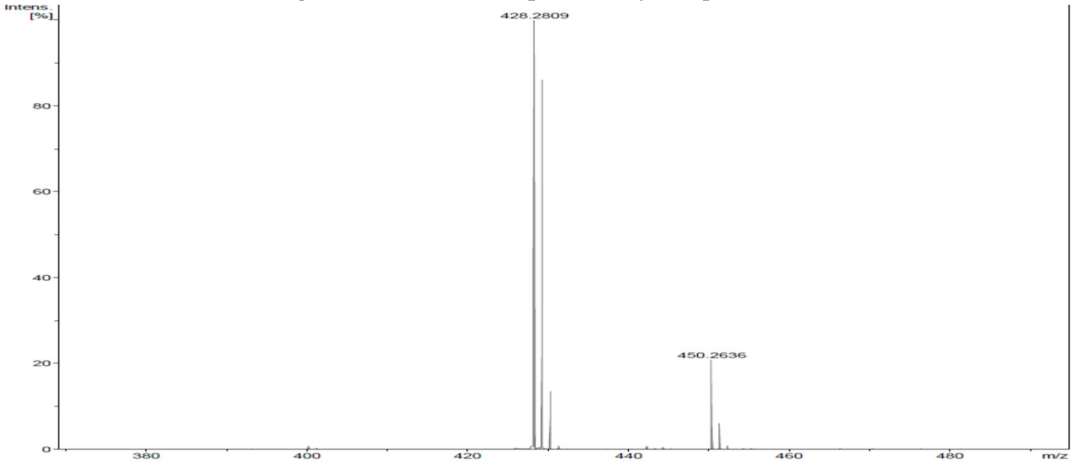

Figure S40. HRMS spectrum of compound ***1n***

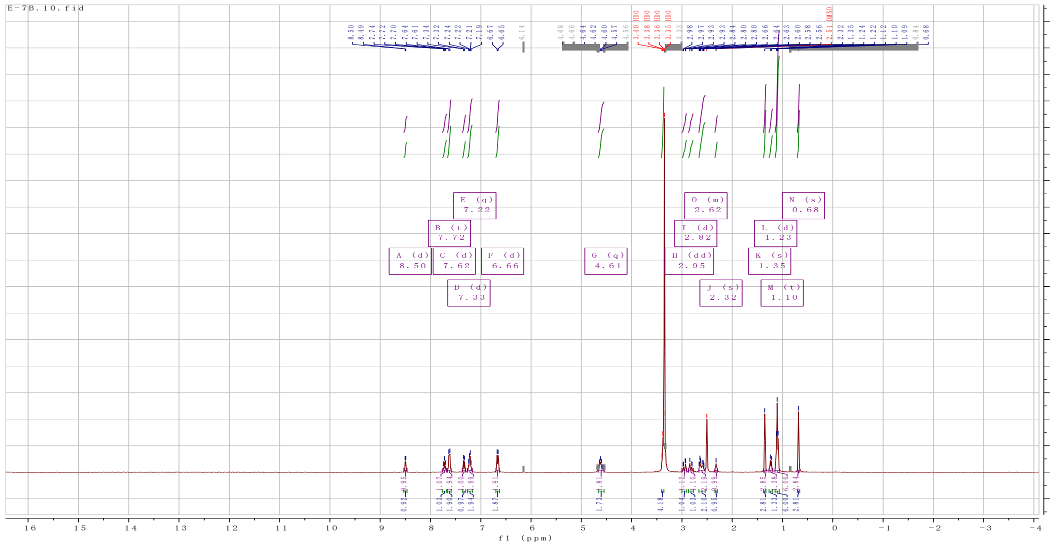

Figure S41. ^{1}HNMR spectrum of compound ***1n***

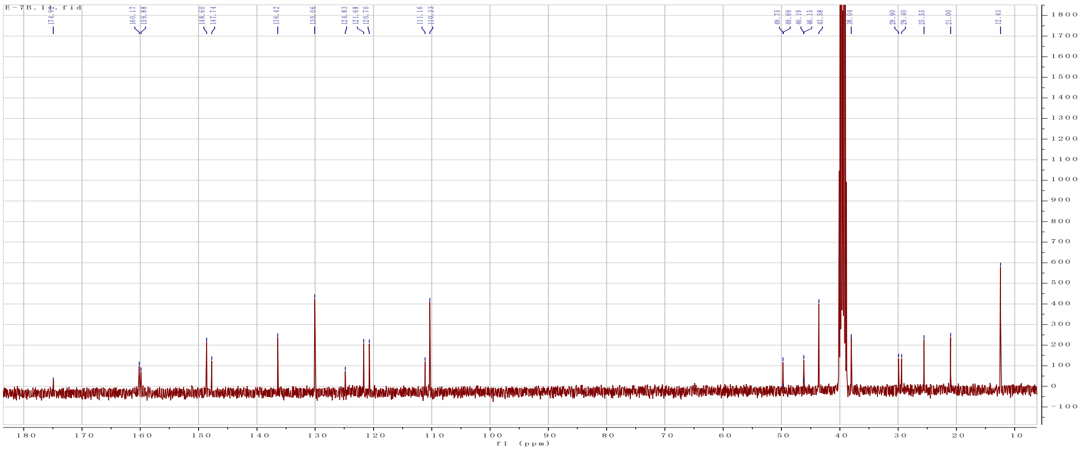

*Figure S42. ^{13}C NMR spectrum of compound **1n***

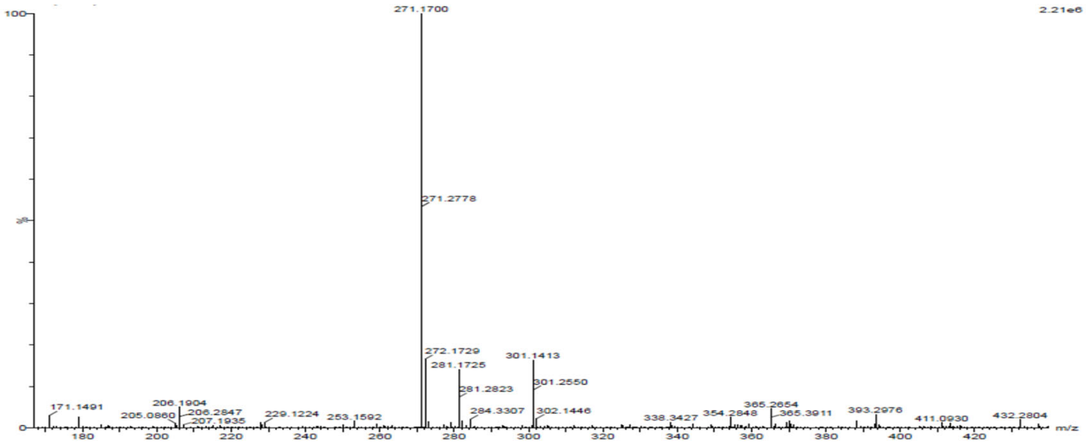

*Figure S43. HRMS spectrum of compound **2a***

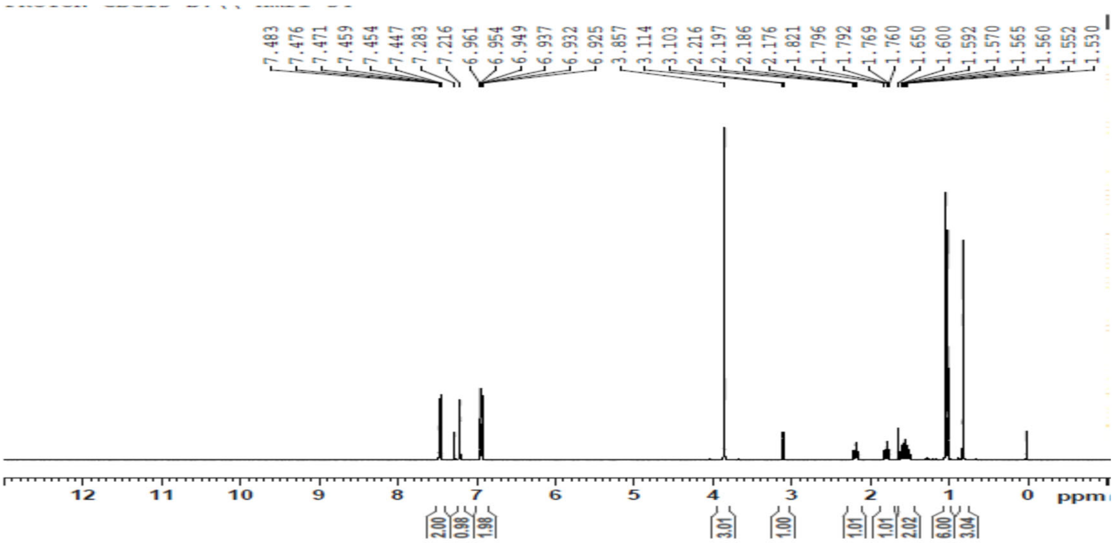

*Figure S44. ^{1}HNMR spectrum of compound **2a***

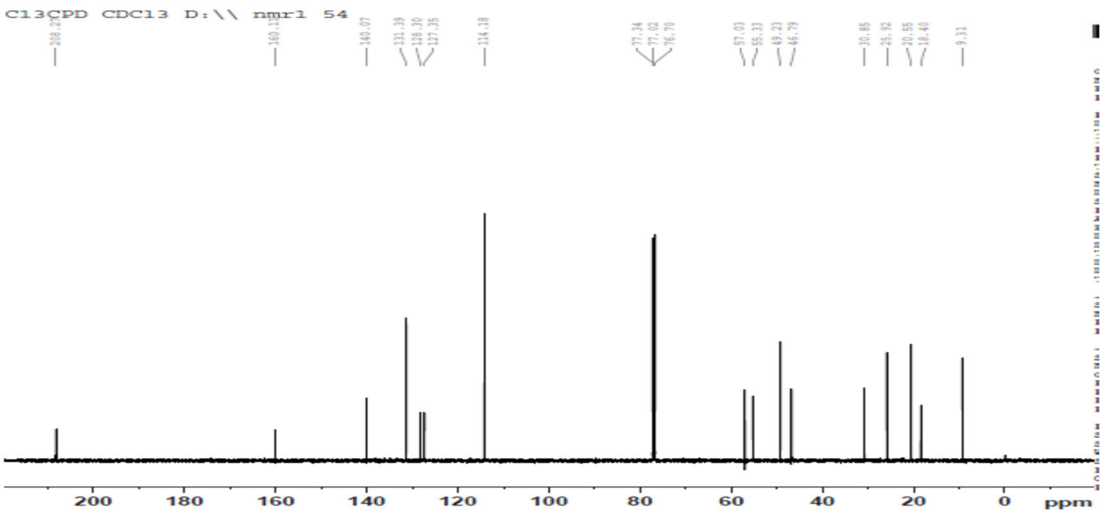

*Figure S45. ^{13}C NMR spectrum of compound **2a***

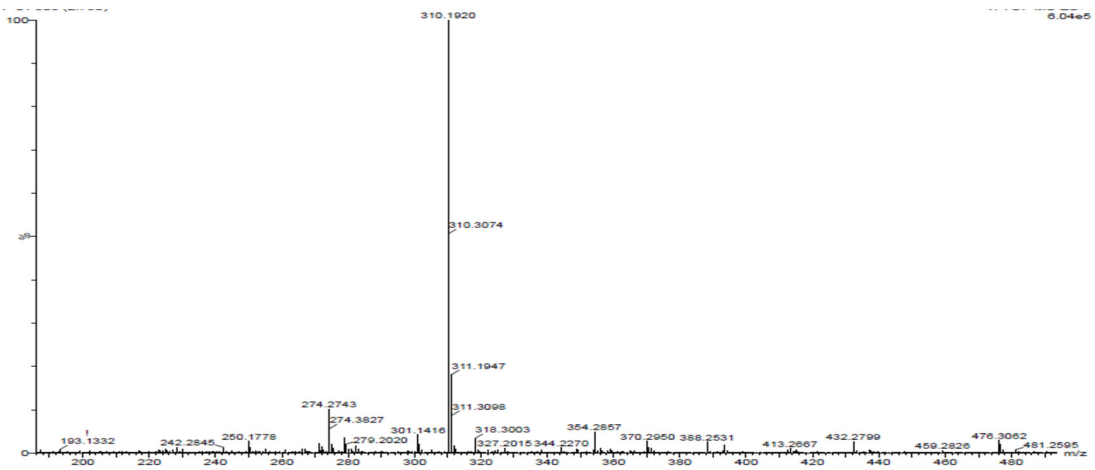

*Figure S46. HRMS spectrum of compound **2b***

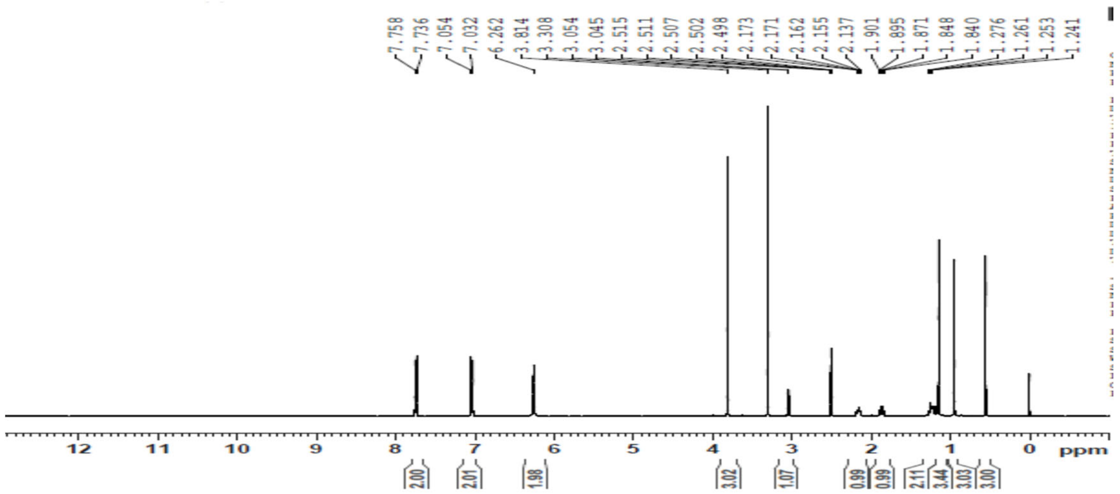

*Figure S47. ^{1}HNMR spectrum of compound **2b***

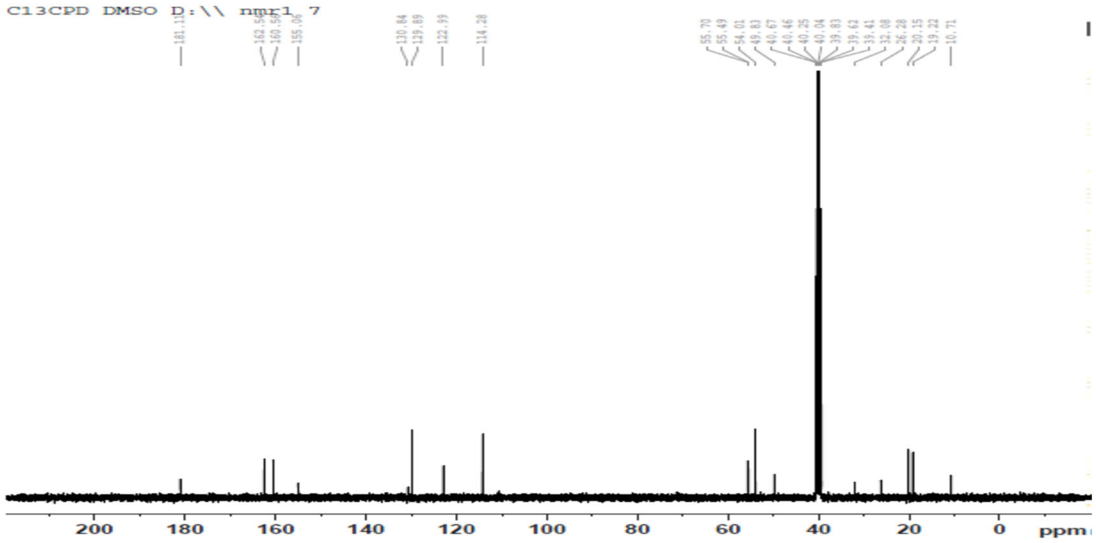

*Figure S48. ^{13}C NMR spectrum of compound **2b***

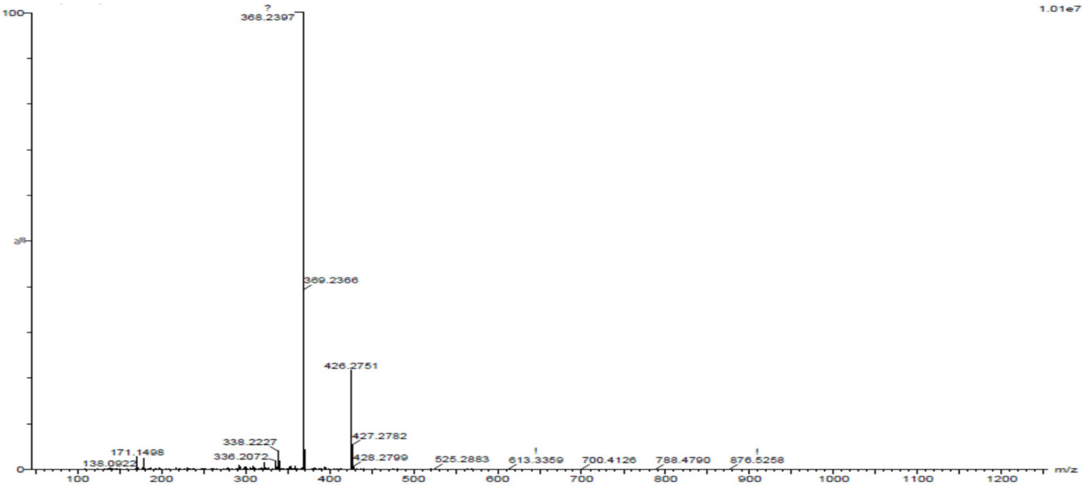

*Figure S49. HRMS spectrum of compound **2c***

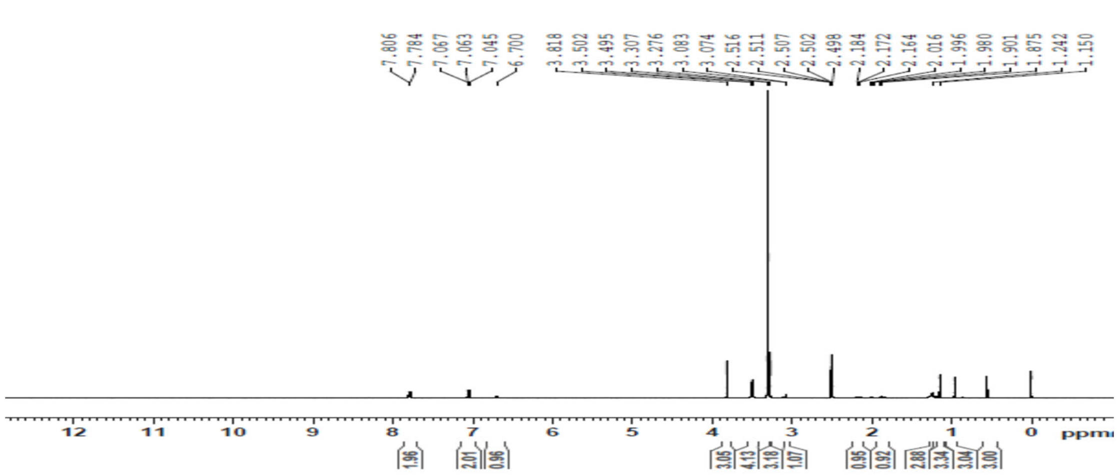

*Figure S50. ^{1}HNMR spectrum of compound **2c***

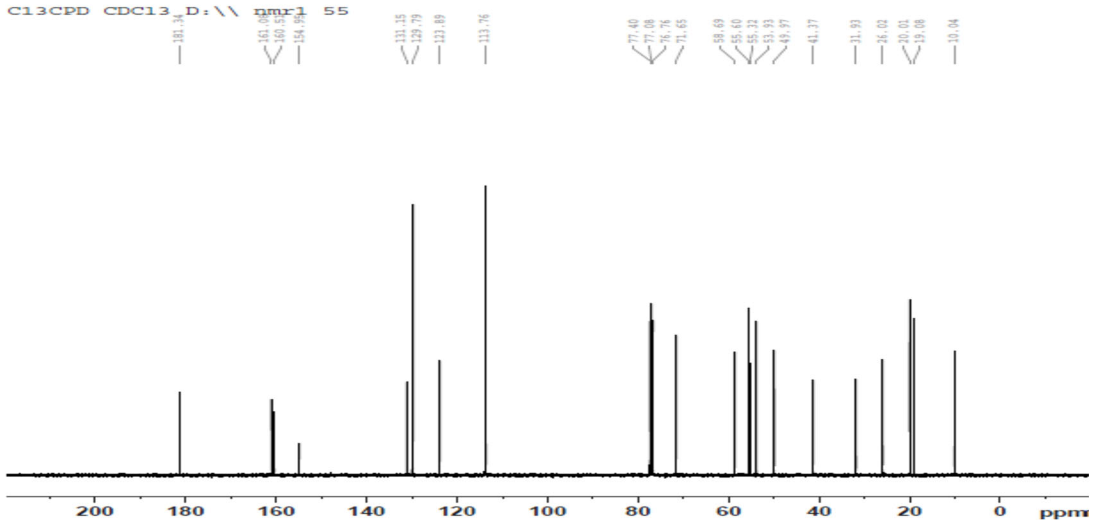

Figure S51. ^{13}C NMR spectrum of compound **2c**

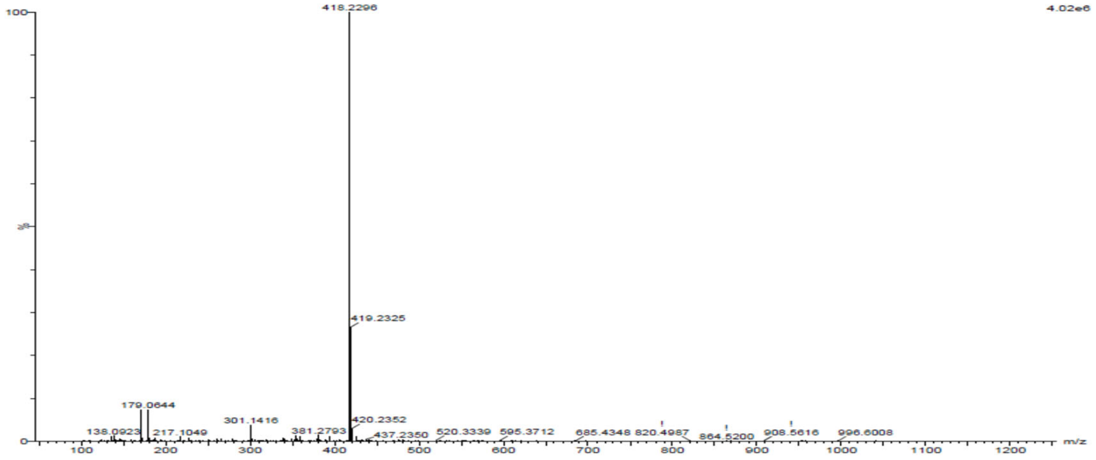

Figure S52. HRMS spectrum of compound **2d**

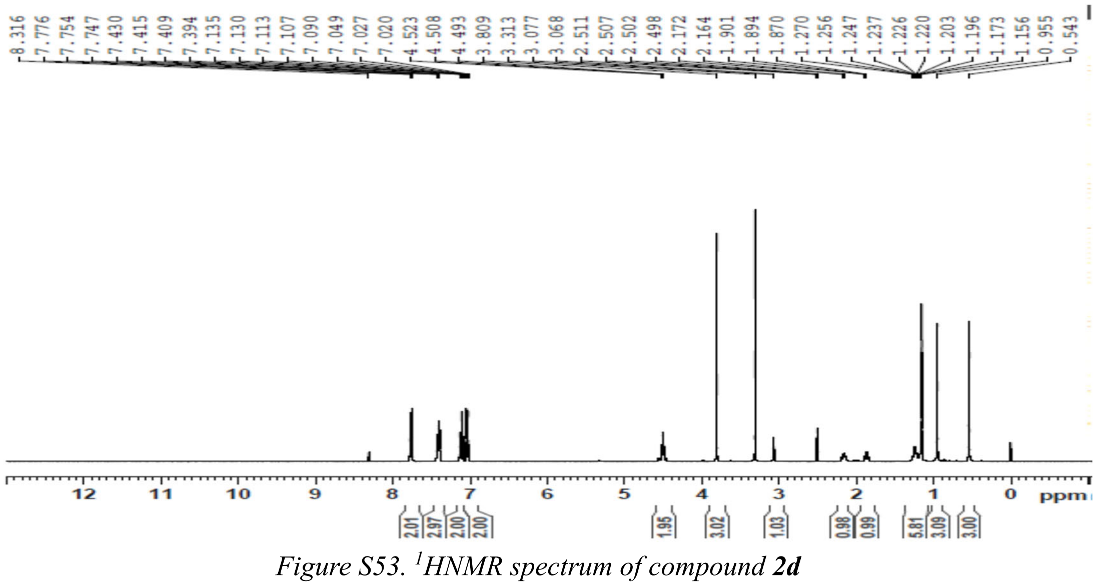

Figure S53. ^{1}HNMR spectrum of compound **2d**

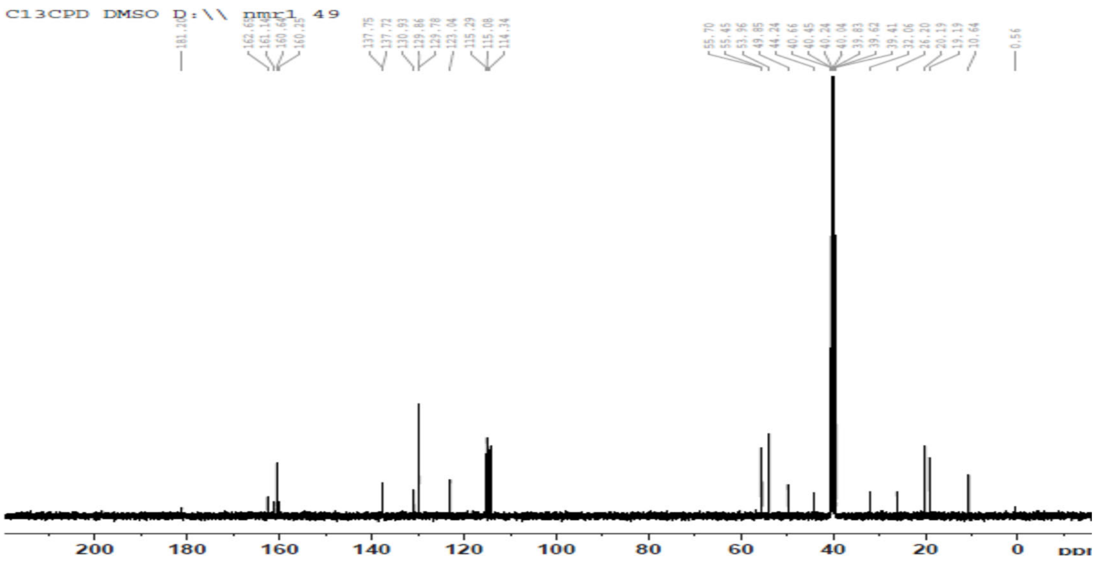

*Figure S54. ^{13}C NMR spectrum of compound **2d***

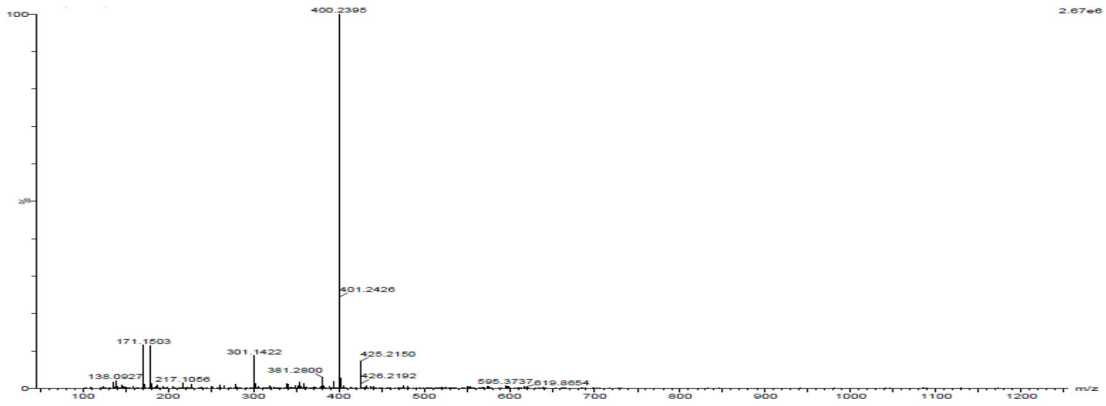

*Figure S55. HRMS spectrum of compound **2e***

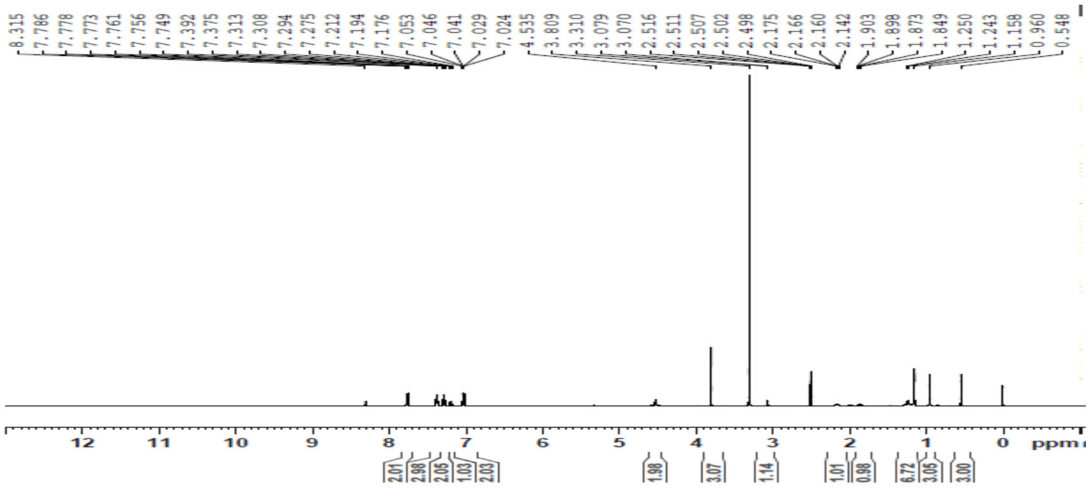

*Figure S56. ^{1}HNMR spectrum of compound **2e***

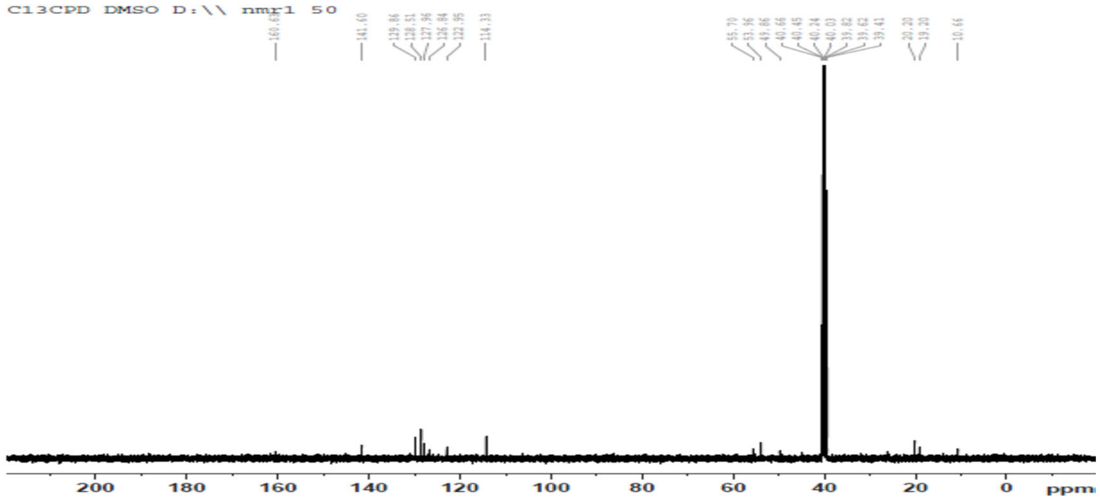

Figure S57. ^{13}C NMR spectrum of compound **2e**

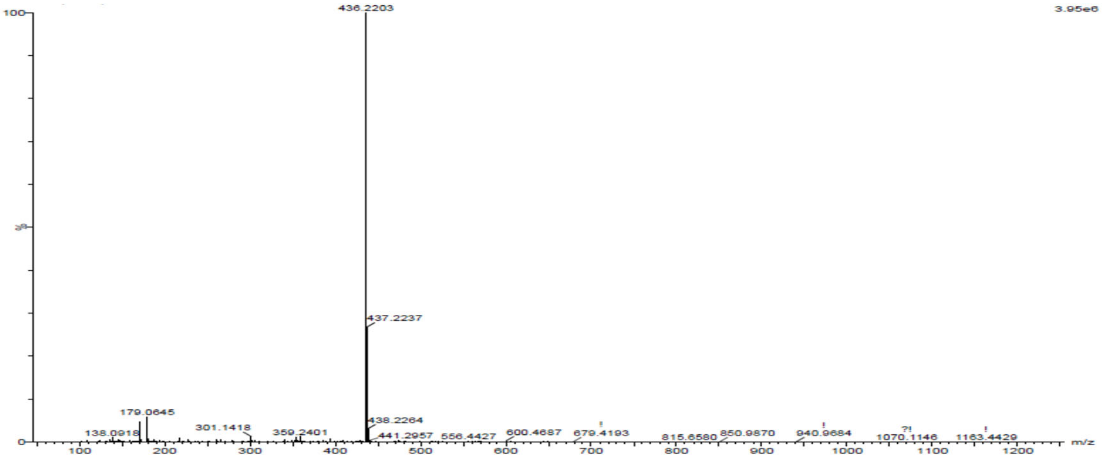

Figure S58. HRMS spectrum of compound **2f**

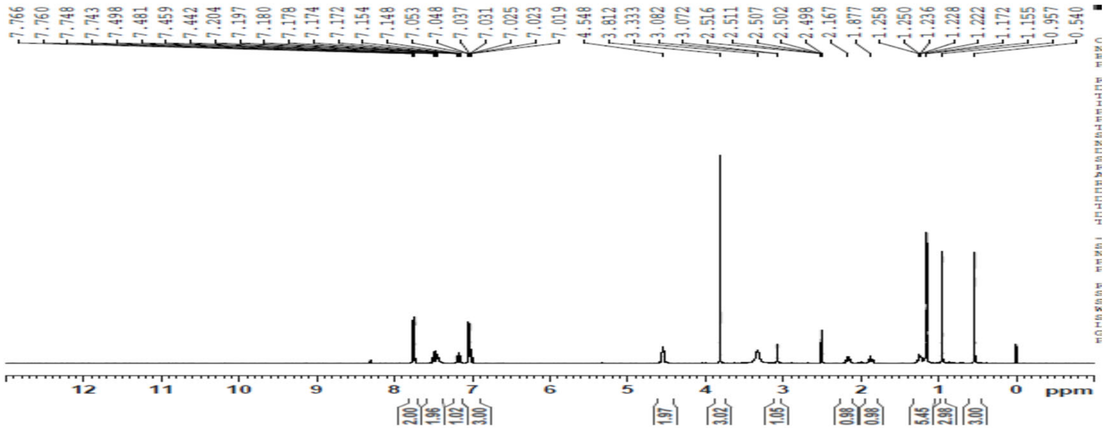

Figure S59. ^{1}HNMR spectrum of compound **2f**

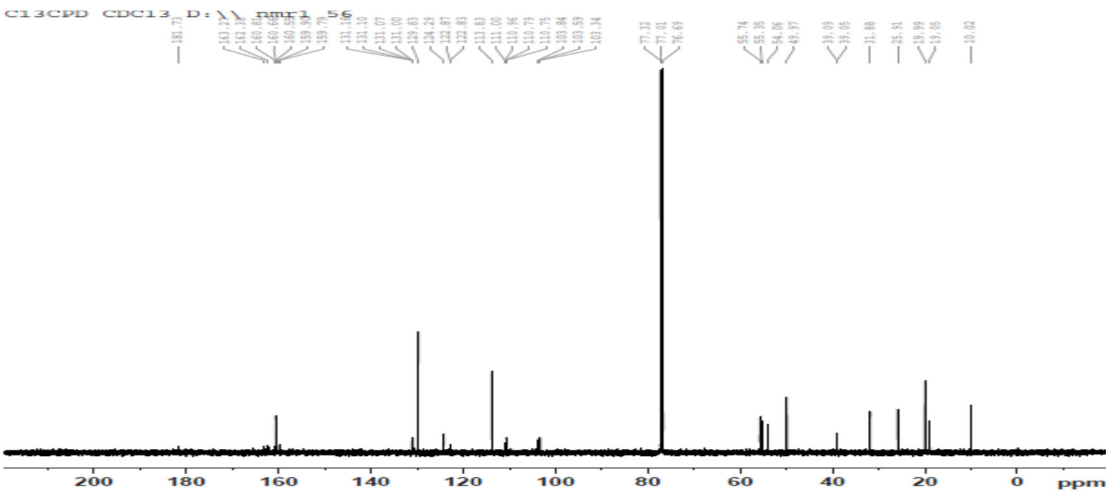

*Figure S60. ^{13}C NMR spectrum of compound **2f***

Table S1 Flexible docking result of **1f** and the standard compound (PDB ID:**2AZ5**)

**Compound**	**PDB**	Binding Residues	Docking Score
**1f**	**2AZ5**	Ser60, Try59	-7.964
**Aspirin**	**2AZ5**	Ser60, Try59	-8.143
